# Nanodrug Delivery Systems in Antitumor Immunotherapy

**DOI:** 10.34133/bmr.0015

**Published:** 2024-04-25

**Authors:** Zishuo Guo, Jinhong Ye, Xuehao Cheng, Tieshan Wang, Yi Zhang, Kaili Yang, Shouying Du, Pengyue Li

**Affiliations:** ^1^ Beijing University of Chinese Medicine, Beijing 102488, China.; ^2^ YiDu Central Hospital of Weifang, Weifang, Shandong 262500, China.

## Abstract

Cancer has become one of the most important factors threatening human health, and the global cancer burden has been increasing rapidly. Immunotherapy has become another clinical research hotspot after surgery, chemotherapy, and radiotherapy because of its high efficiency and tumor metastasis prevention. However, problems such as lower immune response rate and immune-related adverse reaction in the clinical application of immunotherapy need to be urgently solved. With the development of nanodrug delivery systems, various nanocarrier materials have been used in the research of antitumor immunotherapy with encouraging therapeutic results. In this review, we mainly summarized the combination of nanodrug delivery systems and immunotherapy from the following 4 aspects: (a) nanodrug delivery systems combined with cytokine therapy to improve cytokines delivery in vivo; (b) nanodrug delivery systems provided a suitable platform for the combination of immune checkpoint blockade therapy with other tumor treatments; (c) nanodrug delivery systems helped deliver antigens and adjuvants for tumor vaccines to enhance immune effects; and (d) nanodrug delivery systems improved tumor treatment efficiency and reduced toxicity for adoptive cell therapy. Nanomaterials chosen by researchers to construct nanodrug delivery systems and their function were also introduced in detail. Finally, we discussed the current challenges and future prospects in combining nanodrug delivery systems with immunotherapy.

## Introduction

According to the “IARC Biennial Report 2020-2021” published on 2022 January 6 by the International Agency for Research on Cancer, a division of the World Health Organization, 19.29 million new cancer cases and up to 9.23 million deaths occurred in 2020. Cancer involves various subjects such as genetics, cytology, and tissue biology, and it has become the greatest threat to human life and health. Cancerous cells in the human body are characterized by evading apoptosis, sustained angiogenesis, limitless replicative potential, self-sufficiency of growth signals, insensitivity to antigrowth signals and tissue invasion and metastasis [1,2]. With the advancement of human scientific knowledge, the link between cancer and the human immune system is gradually being recognized, and cancer treatment gradually tends to focus on the activation of the autoimmune system to kill cancer cells and control the spread and metastasis of cancer cells [3–6]. The human immune system can recognize tumor cells and eliminate them in time to maintain the stability of internal environment. However, in the process of tumor development, tumor cells have developed various escape mechanisms to evade recognition and killing by the immune system [7,8]. The tumor microenvironment (TME), which is suitable for the growth of tumor cells, is formed through the interaction between tumors and the body’s immune system. TME is composed of extracellular matrix, basement membrane, endothelial cells, tumor cells, and a variety of recruited stromal cells, immune cells, etc. It is characterized by hypoxia, nutrient depletion, immunosuppression, and subacidity and plays an important role in regulating the metastasis and invasion of tumors [9,10]. In TME, tumor cells evade the immune system through multiple immunosuppressive mechanisms [11].

Tumor immunotherapy aims to reverse the immune suppression of TME and activate the autoimmune system through drugs or adoptive cells to achieve tumor treatment [12]. Compared with chemotherapy (CT) and radiotherapy, immunotherapy has the advantages of fewer side effects and higher safety, and more importantly, it can establish long-term immune memory and control the proliferation and metastasis of tumor cells [12,13]. As an emerging mode of cancer treatment, immunotherapy has shown efficacy in the treatment of various cancers, and a large number of clinical studies with immune monotherapy and combination therapy have been carried out in succession [14,15]. Currently, the main approaches of tumor immunotherapy used in clinical practice include cytokine therapy, immune checkpoint blockade therapy (ICB therapy), cancer vaccine, and adoptive cell therapy (ACT therapy). With the deepening of study, the drawbacks of immunotherapy have gradually emerged and have become the major obstacle limiting its clinical application.

Firstly, for cytokine therapy, there are 2 major problems—short half-life and systemic toxicity—that limit its clinical applications. Secondly, for ICB therapy, most patients have a low response to single immunotherapy, with only approximately 10% to 30% of patients responding effectively to immune checkpoint inhibitors [12,16]. In nonimmunogenic tumors (cold tumors) that respond poorly to immunotherapy, T-cell numbers are low, antigen availability is poor, antigen-presenting cells (APCs) are rejected, and immune cells lack effective activation signals [17,18]. Additionally, immunotherapy drugs usually have off-target toxicity, and immune-related adverse events cannot be eliminated [19]. Thirdly, for tumor vaccines, it usually does not have too much toxic side effect, but its long onset time, complex transport process in vivo, and weak immune effect also limit its further development. Finally, for ACT therapy, systemic toxicity greatly restricts its use for tumor treatment.

As a novel drug delivery method, nanodrug delivery systems are increasingly emphasized by researchers [20]. Nanodrug delivery systems refer to drug delivery systems at the nanometer scale (10^−9^ m), which have substantial advantages in controlling the solubility, stability, and accumulation of drugs in cells [21]. Currently, many nanomedicines have been marketed or are undergoing clinical studies with excellent tumor therapeutic effects (Table 1). However, these nanomedicines are mainly intended to improve traditional tumor treatments such as CT and radiotherapy, and nanomedicines for immunotherapy are currently in the stage of animal experiment. Although there are not yet any marketed nanomedicines on tumor immunotherapy, compared with traditional drug delivery methods, the advantages of nanodrug delivery methods in tumor immunotherapy, such as good tissue specificity, high bioavailability, and low systemic toxicity, are increasingly appreciated [22]. As far as marketed nanomedicines are concerned, there are far more nanomedicines prepared by organic nanomaterials than by inorganic nanomaterials, but there are also nanomedicines prepared by inorganic nanomaterials that are undergoing clinical trials. For immunotherapy, different nanomaterials can solve different problems for different immunotherapies. When selecting nanomaterials, researchers need to choose different nanomaterials based on the problem they are attempting to solve.

**Table 1.** Summary table form for nanomedicines marketed or in clinical trials

Drug	Nanocarrier type	Nanocarrier	Product name	Indications	Clinical translation phase
Doxorubicin	Organic	Liposome	Doxil/Caelix	Ovarian cancer; metastatic breast cancer	Approved
Adriamycin	Oorganic	Liposome	Mycoet	Breast cancer	Approved
Daunorubicin	Organic	Liposome	DaunoXome	Kaposissarcoma	Approved
Vincristine	Organic	Liposome	Onco-TCS	Non-Hodgkin lymphoma	Approved
Irinotecans	Organic	Liposome	Onivyde	Metastatic abenocarcinoma	Approved
L-MTP-PE	Organic	Liposome	Mepact	Nonmetastatic osteosarcoma	Approved
Paclitaxel	Organic	Albumin-based NPs	Abraxanc	Breast cancer	Approved
Doxorubicin	Organic	Pegylated liposome	Lipo Dox	Acquired immune deficiency syndrome-related Kaposi's sarcoma	Approved
Paclitaxel	Organic	mPEG-PLA	Genexol-PM	Breast cancer, lung cancer, pancreatic cancer	Approved
Paclitaxel	Organic	Polymeric micelles	Paclical	Ovarian cancer	Approved
Adriamycin	Organic	Polyisocyanoacrylate	Transdrug	Liver cancer	Approved
Adriamycin	Organic	Liposome	Themodox	Liver cancer	Clinical Trial Phase III
Cisplatin	Organic	Liposome	Lipplatin	Non-small cell lung cancer	Clinical Trial Phase III
Paclitaxel	Organic	Liposome	EndoTAG-1	Liver cancer	Clinical Trial Phase III
Paclitaxel	Organic	Polymeric micelles	NK105	Gastric carcinoma	Clinical Trial Phase III
Cisplatin	Organic	Polymeric micelles	NC-6004	Pancreatic cancer	Clinical Trial Phase III
HfO_2_	Inorganic	HfO_2_ nanoparticle	NBTXR3	Head and neck squamous cell carcinoma	Clinical Trial Phase III
Paclitaxel	Organic	Liposome	LEP-ETU	Metastatic breast cancer	Clinical Trial Phase II
SN-38	Organic	Liposome	LE-SN38	Metastatic colorectal cancer	Clinical Trial Phase II
mitoxantrone	Organic	Polybutylcyanacrylate	DHAD-PBCA-NPs	Liver cancer	Clinical Trial Phase II
7-Ethyl-10-hydroxycamptothecin	Organic	Polymeric micelles	NK102	Breast cancer	Clinical Trial Phase II
Irinotecans	Organic	Liposome	IHL-305	Solid tumor	Clinical Trial Phase I
Paclitaxel	Organic	mPEG-b-PDLLA	Nanoxel	Breast cancer	Clinical Trial Phase I
Oxaliplatin	Organic	Polymeric micelles	NC-4016	Solid tumor	Clinical Trial Phase I
Near-infrared (NIR) dye	Inorganic	SiO_2_	Cornell Dots	Melanoma, brain tumor	Preclinical trials
SiO_2_-Au	Inorganic	SiO_2_-Au	AuroLase	Lung cancer	Preclinical trials

**Fig. 1.**
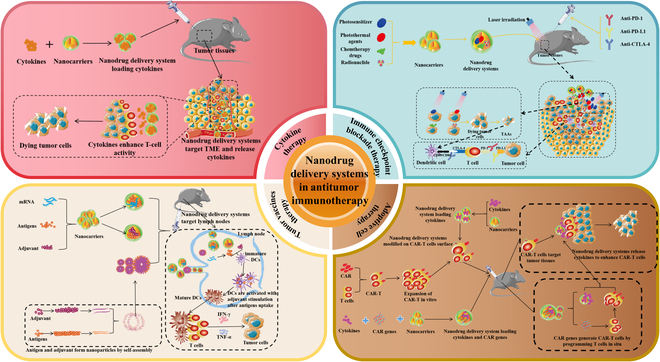
Nanodrug delivery systems employed in cytokine therapy, ICB therapy, tumor vaccines, and ACT therapy to enhance antitumor immune responses.

With the development of nanotechnology, various nanomaterials have been used as carriers of therapeutic drugs according to their different physicochemical properties and have achieved excellent results in tumor treatment [11,23]. Firstly, nano-targeted drug delivery methods can significantly enhance the efficacy of immunotherapeutic drugs, prevent their leakage during transportation in vivo, and prevent the off-target effects and systemic toxicity. This property of nanodrug delivery systems could help cytokine therapy solve the problem of short half-life. Secondly, nanodrug delivery systems can not only passively target tumor tissues through the enhanced permeation and retention effect but also modify specific ligands and antibodies on their surface to actively target TME [19,20,24,25]. This precisely targeted feature could help cytokine therapy and programmed cell death protein 1 (PD-1)/programmed cell death 1 ligand 1 (PD-L1) antibody in ICB therapy solve the problem of systemic toxicity and help tumor vaccines solve the problem of complex transport in vivo. Thirdly, rational design of nanodrug delivery systems can achieve controlled release of loaded drugs (e.g., pH-responsive release, etc.) and improve the utilization of therapeutic drugs. This could not only solve the problem of systemic toxicity of cytokine therapy and ACT therapy but also help to solve the problem of weak immune effect of tumor vaccine therapy. Fourthly, some nanomaterials have adjuvant properties themselves, which can improve the effect of immunotherapy and can solve the problem of low immune effect for ICB therapy, tumor vaccine therapy, or ACT therapy [26]. Finally, nanodrug delivery systems provides a biocompatible and safe drug delivery platform for combining CT, radiotherapy, and other therapeutic modalities with immunotherapy to synergistically amplify the immune response and improve the effectiveness of tumor treatment. In this review, we summarized the application examples of nanodrug delivery systems in 4 immunotherapy treatments: cytokine therapy, ICB therapy, tumor vaccines, and ACT therapy and discussed how different nanomaterials contribute to different immunotherapy treatments and amplify the immune effect (Fig. 1). In addition, in the discussion section, we summarized the advantages and disadvantages of different nanomaterials for different immunotherapies and discussed the challenges of clinical translation for antitumor immune nanomedicines.

## The Application of Nanodrug Delivery Systems in Cytokine Treatment

Cytokines are a class of biologically active molecules mainly produced by immune cells that play an important role in maintaining physiological immune homeostasis and regulating immune responses, and make a important contribution to mediating the immune response of immune and nonimmune cells in the TME. Cytokines participate in regulating the proliferation, differentiation, and effector functions of immune cells, such as stimulating the survival, proliferation, and functions of T cells and natural killer cells (NK cells), and are deeply involved in the antitumor immune process [27,28].

Currently, many types of cytokines have been used in clinical trials with some effective results, but the delivery of cytokines faces certain obstructions due to their narrow therapeutic range and short half-life in vivo [29]. Cytokines commonly used for antitumor treatment include interleukin-2 (IL-2), interleukin-12 (IL-12), and interleukin-15 (IL-15) from the interleukin family, and tumor necrosis factor-α (TNF-α) from the tumor necrosis factor superfamily. IL-2 was the first interleukin approved for the treatment of tumors. It promotes the proliferation and effector killing of leukocytes but has serious side effects (for example, pulmonary edema) and an extremely short half-life in human serum (only 5 to 7 min) [30]. In antitumor immune response, IL-2 is helpful in maintaining T-cell function and controlling the growth of tumors, and studies have shown that the number of CD8^+^ T cells is significantly reduced after blocking the IL-2 receptor [31]. However, IL-2 has shown protumor activity as it preferentially stimulates regulatory cells (Tregs) and promotes the proliferation of Tregs, which is detrimental to clinical antitumor therapy [32]. IL-12 is mainly produced by APCs such as macrophages and dendritic cells (DCs), which can activate T cells and NK cells and promote T helper 1(Th1)-mediated adaptive immunity, but has limitations in clinical application such as a short half-life in vivo, weak tumor targeting, and severe host toxicity [27,30]. IL-15 belongs to the IL-2 family and has similar immunostimulatory functions to IL-2. Notably, IL-15 does not lead to Treg expansion, but its short half-life and insufficient in vivo efficacy limit its use [33]. In addition, IL-15 can maintain the survival and development of NK cells [34]. TNF-α is a type of proinflammatory cytokine secreted by macrophages and monocytes involved in normal inflammatory and immune responses. TNF-α can directly induce apoptosis of tumor cells, but it causes serious systemic side effects such as fever when injected [35].

**Fig. 2.**
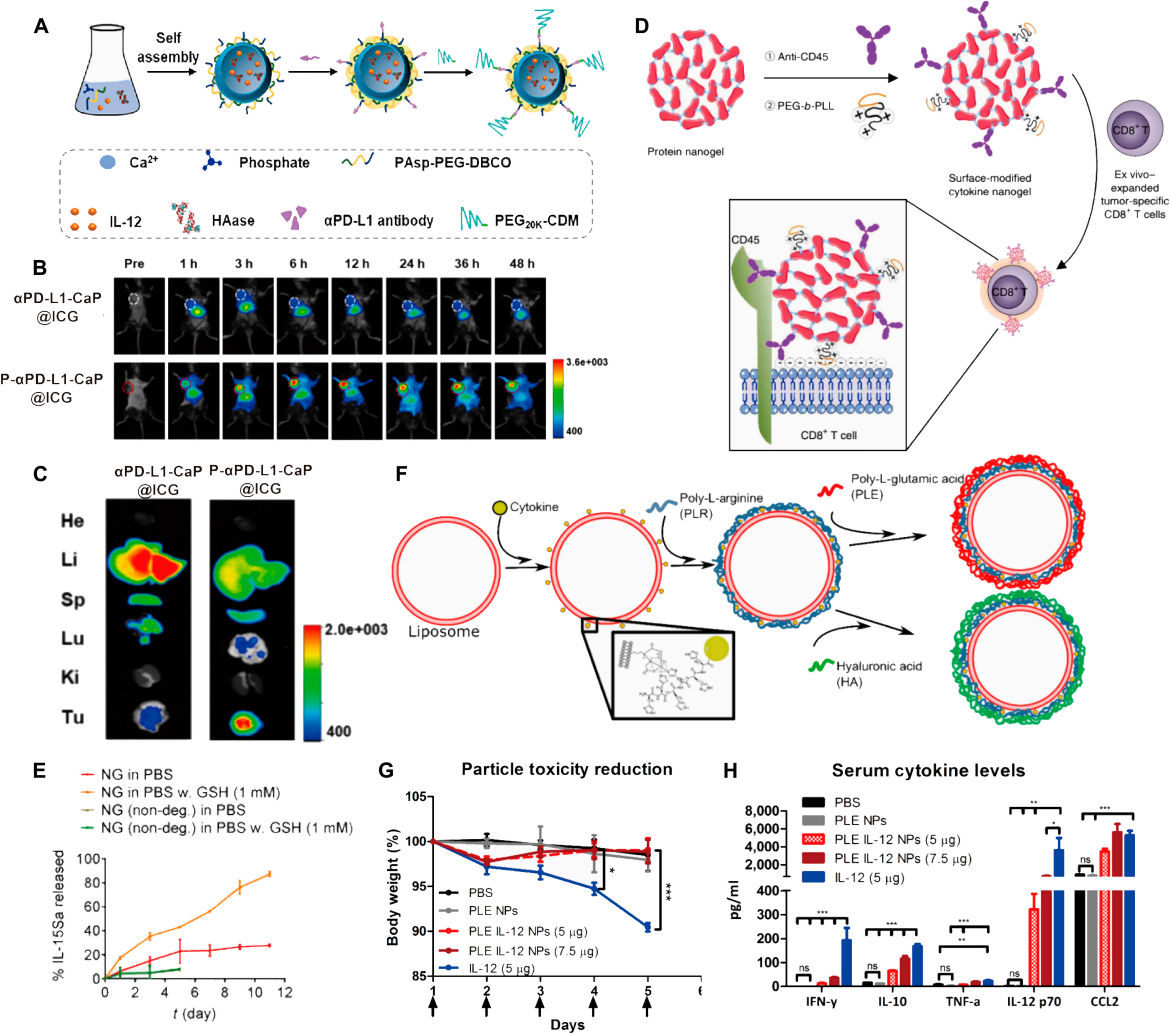
Nanodrug delivery systems in cytokine therapy. (A) Schematic illustration of the preparation for P-αPD-L1-CaP@H/I nanodrug. (B) In vivo fluorescence images of Hepa1 to Hepa6 tumor-bearing mice receiving intravenous injection of αPDL1-CaP@ICG and P-αPD-L1-CaP@ICG at different time points. (C) Ex vivo fluorescence images of major organs (He, heart; Li, liver; Sp, spleen; Lu, lung; Ki, kidney) and tumors (Tu) collected 48 h after nanodrug injection. Adapted with permission from [37], copyright 2023 Journal of Controlled Release. (D) Scheme for surface modification of cytokine-NGs to facilitate efficient and stable anchoring on T cell surfaces. (E) Release kinetics of cytokines from redox-responsive or nondegradable IL-15Sa-NGs in phosphate buffered saline (PBS) with or without added glutathione (GSH) as a reducing agent. Adapted with permission from [39], copyright 2018 Nature Biotechnology. (F) Schematic of layer-by-layer buildup of particle and cytokine attachment. (G) Body weight change (mean ± standard error of the mean [SEM]) of healthy animals treated as indicated subcutaneously (PLE IL-12 NPs 5 μg *n* = 5, *n* = 3 all other groups). *indicates *P* < 0.05, ***indicates *P* < 0.001 as measured by 2-way analysis of variance (ANOVA) with Bonferroni post hoc test across all groups. (H) Cytokine response (mean ± SEM) in serum taken after dose 5 from (B) as measured by multiplexed assay. *indicates *P* < 0.05, **indicates *P* < 0.01, ***indicates *P* < 0.001 as measured by 1-way ANOVA on individual cytokine groups with Bonferroni post hoc test comparing all groups to IL-12 (5 μg) and PBS to PLE NPs. Adapted with permission from [40], copyright 2020 ACS Nano.

Cytokines must reach sufficient concentrations in the TME to induce a strong antitumor immune response in vivo, but this usually results in severe systemic toxicities. Short half-life of cytokines leads to their inability to circulate in the body for a long time. One of the strategies to solve these problems is to employ nanodrug delivery systems to load cytokines [36]. Currently, researchers mainly focus on using nanodrug delivery systems to solve the following problems: (a) Loading cytokines to target tumor tissues to reduce the systemic toxicity; and (b) Controlling cytokine release to overcome the short half-life. Nanodrug delivery systems can deliver cytokines to tumor tissues and release them continuously, which allows tumor treatment with less damage to other healthy tissues. Xiao et al. [37] prepared a loaded IL-12 nanodrug delivery systems (P-αPD-L1-CaP@H/I) by using a pH and matrix metalloproteinase-2 (MMP-2) dual-sensitive polymer/calcium phosphate (CaP) hybrid nanocarrier and adding tumor acidity-sheddable polyethylene glycol (PEG) on its surface (Fig. 2A). P-αPD-L1-CaP@H/I accurately targeted tumor tissues and reduced accumulation in other tissues and organs to decrease the systemic toxicity from IL-12 (Fig. 2B and C). In addition, nanoparticles (NPs) modified with polymers such as serum proteins or PEG significantly increased the circulating half-life of cytokines [38]. Tang et al. [39] delivered the IL-15 superagonist IL-15SA by using protein nanogel material to achieve controlled release regulation of cytokine-like drugs. This approach is based on a synthetic disulfide-containing bis-N-hydroxy succinimide (NHS) cross-linker (NHS-SS-NHS) loaded with IL-15SA that is stably maintained stable on the NP surface by anti-CD45 and PEG-b-PLL modifications to increase the retention time on the T-cell surface (Fig. 2D). The IL-15SA-loaded nanogel lyses during the reduction reaction of T cells and releases the drug to achieve the controlled release of cytokines (Fig. 2E). The controlled release of cytokines not only solved the shortcoming of cytokines that cannot be retained in the body for a long time but also improved the immune activation effect. In another example by Barberio et al. [40], an IL-12 delivery nanoparticle (PLE-IL-12-NPs) was designed by layer-by-layer technology, which greatly reduced the off-target toxicity of IL-12 (Fig. 2F). Single-chain IL-12 was attached to the surface of the nanoliposomes, and the nanoliposomes were modified by a polyelectrolyte bilayer, which allowed the targeting of the nanodrug delivery system while shielding IL-12 from toxicity and improving safety. Toxicity test results showed that PLE-IL-12-NP significantly reduced IL-12-induced systemic toxicity (Fig. 2G and H).

In cytokine therapy applications, researchers have increasingly used organic nanomaterials or hybrids of organic and inorganic materials to construct nanodrug delivery systems (Table 2). Firstly, compared to inorganic nanomaterials, organic nanomaterials such as PEGs and lipid nanoparticles (LNPs) have better compatibility with cytokines, which can keep cytokines’ higher activity during the transportation process. Secondly, although NPs prepared from organic nanomaterials have low drug loading capacity, the microefficient character of cytokine therapy drugs allows nanodrug delivery systems to provide powerful antitumor effect even with little cytokine loading. Finally, most cytokines target immune cells for action, so researchers prefer to use organic nanomaterials with better histocompatibility to load cytokines. Cytokines plays a important role in antitumor immunotherapy but are currently under-researched clinically due to their short in vivo half-life. Nanodrug delivery systems can help cytokines accurately target tumors or related immune cells, extend their retention time in vivo, and reduce the toxicity and side effects of cytokines [37].

**Table 2.** Nanodrug delivery systems in cytokine therapy

Nanomaterials type	Nanocarrier	Load treatment drugs	Cancer model	Immune cell type	Therapeutic outcome	References
Organic and inorganic	Polymer/calcium phosphate (CaP) hybrid	IL-12	Hepa1 to Hepa6 cells	T cells	Induced a robust antitumor immunity for efficiently suppressing HCC growth in mice	[37]
Organic	Protein nanogels	IL-15 superagonist complex	B16F10	T cells	Substantially increased tumor clearance by murine T cell and human CAR-T cell therapy in vivo	[39]
Organic	Liposome	IL-12	MC38	T cells	Showed efficacy against a tumor challenge in both colorectal and ovarian tumors	[40]
	Hyaluronic acid		HM-1			

## Application of Nanodrug Delivery Systems in ICB Therapy

The immune checkpoint pathway is an important way for tumor cells to suppress the antitumor immune response and escape the surveillance and killing of the immune system during tumor development [41]. PD-1/PD-L1 and cytotoxic T lymphocyte-associated antigen-4 (CTLA-4) are 2 common escape pathways. PD-1 is a protein expressed on the surface of activated T cells; when PD-1 is combined with PD-L1/PD-L2 expressed by tumor cells, it will inhibit the immune response of T cells [42]. In addition, the binding of PD-1 on cytotoxic T cells to PD-L1 ligands on tumor cells accelerates the growth of tumors [43]. CTLA-4 is a receptor expressed on the surface of T cells that inhibits T-cell activation and plays an important role in maintaining immune homeostasis in vivo. However, when it binds to 2 ligands, CD80 and CD86, it decreases the killing activity of T cells against tumor cells [44,45]. CTLA-4 also competes with the costimulatory molecule CD28 on the surface of T cells for ligands and inhibits T-cell proliferation [46].

ICB therapy aims to activate the autoimmune system, especially T cells in vivo to recognize and kill tumor cells by blocking the immune checkpoint pathway. The key to ICB treatment is to activate CD8^+^T cells to kill tumor cells. CD8^+^ cytotoxic T cells are the main cytotoxic effector cells that kill tumor cells through the granulocytic pathway, the FasL apoptotic pathway, and the secretion of interferon-γ (IFN-γ) and granzyme B [47]. PD-1/PD-L1 blockade therapy and CTLA-4 blockade therapy both enhance the killing effect of the immune system on tumor cells by activating CD8^+^T cells [36]. At present, the drugs used to block the PD-1/PD-L1 pathway, anti-PD-1 nivolumab and pembrolizumab, anti-PD-L1 atezolizumab, avelumab and durvalumab, and anti-CTLA-4 ipilimumab, have been approved by the Food and Drug Administration (FDA) [42,48,49].

The clinical application of ICB therapy can directly interfere with the escape and immunosuppression mechanisms of tumor cells and activate the function of T cells. However, most patients do not respond to ICB therapy, especially those with tumors without immune infiltration [41,50]. Before reaching the TME to perform their function, ICB therapeutic drugs need to cross multiple barriers with appropriate concentrations and low toxicity to ensure a good antitumor effect. The half-life and durability of most immune checkpoint inhibitors (such as ipilimumab) are limited, and there is no guarantee that they can achieve the expected effect after reaching the TME. In the process of reaching the immune checkpoint, drugs may lead to overactivation of the immune system and autoimmune dysregulation [51]. PD-1/PD-L1 immune checkpoint inhibitors have relatively few side effects but still suffer from adverse immune system reactions and immune tolerance [16,49].

### Application of nanodrug delivery systems in ICB therapy

**Fig. 3.**
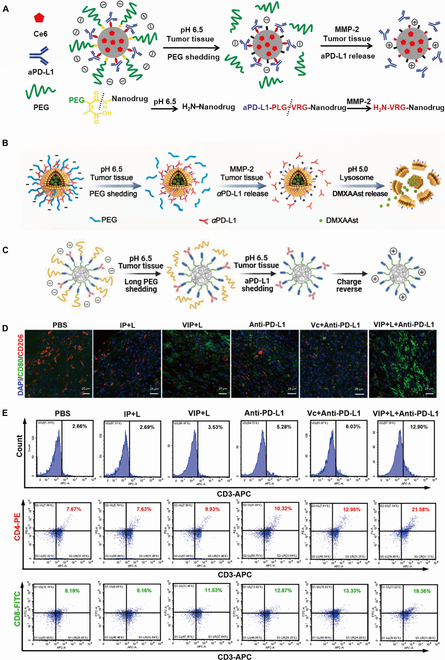
Application of nanodrug delivery systems in immune checkpoint blockade therapy. (A) Schematic illustration of the sensitivity and in vivo performance of the pH and MMP- 2 dual-sensitive PEG-coated nanodrug PEG-CDM-aPD-L1/Ce6 abbreviated as P-aPD-L1/C for tumor-targeting immuno-sonodynamic combination therapy of cancer. Adapted with permission from [57], copyright 2021 Biomaterials. (B) PEG-sheddable nanodrug PEG-LP@αPD-L1/ DMXAAst (P-LPD) with pH and MMP-2 dual sensitivity for codelivery of αPD-L1 and STING activator DMXAAst. Adapted with permission from [58], copyright 2022 Nano Today. (C) Tumor acidity-triggered shedding of both long-chain PEG and aPD-L1 from nanodrug. Adapted with permission from [59], copyright 2023 Acta Biomaterialia. (D) Multiplex immunohistochemical images of CD80^+^ and CD206^+^ in tumor tissues after different treatments. (E) Flow cytometry analysis of CD3^+^, CD4^+^, and CD8^+^ T cell infiltration in tumor tissues for each mouse group: APC antimouse CD3, PE (phycoerythrin) antimouse CD8, and FITC antimouse CD4. FITC, fluorescein isothiocyanate. Adapted with permission from [60], copyright 2022 ACS Nano.

To overcome the limitations of the immune checkpoint inhibitors mentioned above, nanodrug delivery systems have been applied in immunotherapy and have achieved excellent therapeutic results. Firstly, the superior targeting ability of nanodrug delivery systems can help ICB therapeutic drugs target tumors precisely, and they can target tumor tissues through the enhanced permeation and retention effect or modification on the surface of NPs to improve the drug delivery efficiency and therapeutic effect [52]. In addition, nanodrug delivery systems can also carry drugs across some physiological tissues (such as the blood–brain barrier) to achieve ICB therapeutic drug delivery. For example, Wang et al. [53] employed the choline analog methyl N-phneyl carbamate (MPC) as a nanocarrier loaded with anti-PD-1 to cross the blood–brain barrier for immunotherapy against glioma. In addition to targeting tumor cells, nanodrug delivery systems can also target immune cells. Schmid et al. [54] achieved targeted delivery of transforming growth factor β (TGFβ) signaling inhibitors and Toll-like receptor 7/8 (TLR7/8) agonists to PD-1-expressing T cells by modifying antibodies on the surface of poly(lactic-co-glycolic acid) (PLGA)/PEG NPs. This strategy greatly decreased the concentration of drug or antibody and reduced the toxicity while ensuring the efficacy of treatment. Secondly, drug delivery through nanodrug delivery systems can prevent the degradation of drugs by enzymes in the human body, and it can also prolong the residence time of drugs in the circulatory system [55]. Reducing antibody disclosure by modifying PEG on the surface of nanodrug delivery systems is a common approach used by researchers. Wang et al. [56] wrapped the PD-L1 antagonist in the NPs by self-assembly and prevented the drug degradation in vivo through a PEG protective layer on the surface. When the NPs reached the melanoma, the PEG protective layer on the surface was destroyed by MMP-2 overexpressed in the melanoma and released the PD-L1 antagonist, which significantly improved the immunotherapeutic effect in combination with the inhibitor of apoptosis proteins (IAP) antagonist AZD5582 without severe toxic side effects. Huang et al., Guo et al., and Wang et al. [57–59] have also employed this strategy to reduce anti-PD-L1 (aPD-L1) leakage and significantly reduce aPD-L1-induced immune-related adverse effects (Fig. 3A to C). Thirdly, the combined application of nanodrug delivery systems and immunotherapy can amplify the antitumor effect of ICB and enhance the systemic antitumor immune effect [16,55]. For example, Ma et al. [60] prepared a kind of NP, Vc-ICG@LP NPs (VIP) by using PEG-PLGA loaded with vitamin C and indocyanine green (ICG), which could improve the immune response of the TME. After the combination of VIP and anti-PD-L1 therapy, the expression of CD206, a marker of tumorigenic M2-type macrophages, was significantly down-regulated in TME, and CD80, a marker of antitumor M1-type macrophages, was significantly up-regulated (Fig. 3D), suggesting that the combination therapy promoted the transformation of macrophages from the M2-type to the M1-type, which contributed to enhancing the immune infiltration in TME. When vitamin C was combined with anti-PD-L1, the percentage of CD4^+^ T cells in tumors increased from 7.67% to 12.95% and that of CD8^+^ T cells from 8.19% to 13.33%. When the combination was mediated by nanodrug delivery systems, the percentages of CD4^+^ T cells and CD8^+^ T cells increased to 21.58% and 19.36%, respectively, and the infiltration percentage was significantly increased (Fig. 3E). In addition, TLRs are important targets for stimulating the immune system and supporting T-cell activation, and nanodrug delivery system-mediated codelivery of TLR agonists and ICB therapeutic drugs can significantly amplify the ICB-induced systemic immune response. Li et al. [61] prepared TME-targeted NPs by self-assembly of Zn^2+^ with pIC (polycytidylic aid), which was able to activate the TRL3 pathway and significantly enhance the tumor therapeutic effect of αPD-L1: the percentage of CD8^+^ T cells, DCs, and macrophages in tumors was significantly increased. Yu et al. [62] developed a PD-L1/TLR7 dual-targeted nanoantibody-conjugated drug that showed stronger antitumor activity by codelivery of anti-PD-L1 with a TLR7 agonist: The infiltration of CD4^+^ T cells, CD8^+^ T cells, and NK cells in tumors was significantly increased, enhancing the antitumor immune response mediated by anti-PD-L1. Finally, nanodrug delivery systems can be used as a platform to combine immunotherapy with other therapeutic approaches such as photodynamic therapy (PDT), photothermal therapy (PTT), CT, and radiation therapy (RT) to improve tumor treatment efficacy and amplify systemic antitumor immune responses, and we will describe these approaches below.

### Nano-PDT drug delivery systems combined with ICB therapy to amplify antitumor immune response

PDT plays an antitumor role mainly through the generation of reactive oxygen species (ROS) by photosensitizers during the antitumor process. Photosensitizers react with oxygen to produce ROS and singlet oxygen species (^1^O_2_) by absorbing specific wavelengths of light (near-infrared [NIR] light). Both ROS and ^1^O_2_ can selectively induce tumor cell apoptosis without damaging other normal cells [63,64]. PDT also provides a large amount of tumor-associated antigens (TAAs), which triggers immunogenic cell death (ICD) effects and induces antitumor immune responses in vivo [64]. However, when used in clinical practice, PDT has limitations such as insufficient tumor targeting ability, a hypoxic TME in tumor tissue that limits the production of ROS, difficulty in light reaching deep tissues, and poor stability of photosensitizers [65].

**Fig. 4.**
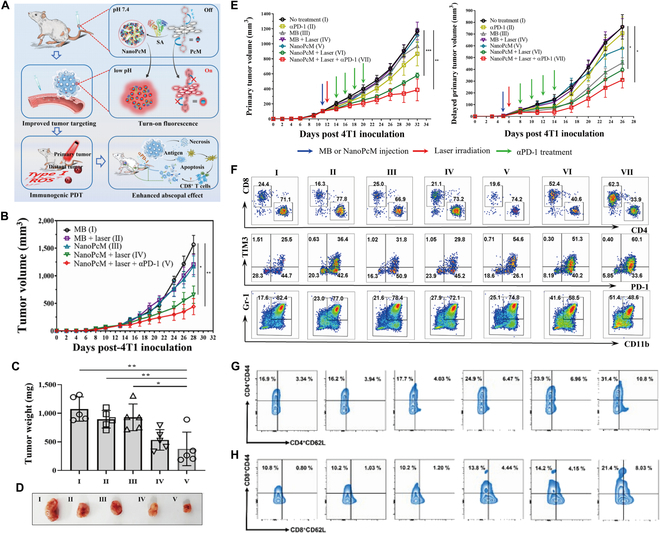
Nano-PDT drug delivery systems combined with ICB therapy to amplify antitumor immune response. (A) Schematic illustration of the structure of NanoPcM, the mechanism of its tumor targeted fluorescence turn-on imaging, and the synergistic antitumor effect caused by NanoPcM based PDT and αPD-1-based immunotherapy. (B) Tumor growth curves in BALB/c mice after NanoPcM injection (*n* = 5 for each group). NanoPcM was injected intravenously when the tumor size reached ∼100 mm^3^, and 24 h later, the tumors were irradiated with a 655-nm laser for 10 min at a power density of 0.2 W/cm2. The αPD-1 antibody was intravenously injected a total of 4 times every 2 d, starting from 24 h after laser irradiation. (C) The tumor weight was measured after resection at the end of the study. (D) Representative photographs of tumor tissue from each group at the end of the study. (E) Tumor growth curves of 4T1primary tumors and 4T1 delayed primary tumors (on the opposite side). (F) Flow cytometric analysis of infiltrating leukocytes from delayed primary tumor tissues excised at the end of the study. Relative proportions of CD8^+^ T cells (CD3^+^CD4^−^ CD8^+^) versus CD4^+^ T cells (CD3^+^CD4^+^ CD8^−^) among CD3^+^ T cells (top panel); exhausted/activated T cells (PD-1^+^ TIM3^−^ PD-1^−^ TIM3^+^, andPD-1^+^ TIM3^+^) among CD8^+^ T cells (middle panel); and MDSCs (CD11b^+^ Gr-1^+^) among total leukocytes (bottom panel). Adapted with permission from [66], copyright 2022 ACS Nano. (G and H) The determination of effector memory cell (CD45^+^CD3^+^CD4^+^CD44^+^CD62-and CD45^+^CD3^+^CD8^+^CD44^+^CD62-) and Tcms (CD45^+^CD3^+^CD4^+^CD44^+^CD62^+^and CD45^+^CD3^+^CD8^+^CD44^+^CD62^+^) in CD8^+^ T cells and CD4^+^ T cells in spleens. Data are presented as mean ± SD. Statistical significance was calculated via 1-way ANOVA with Tukey’s posttest. *indicates *P* < 0.05; **indicates *P* < 0.01; ***indicates *P* < 0.001; ****indicates *P* < 0.0001. Adapted with permission from [67], copyright 2022 Advanced Materials.

At present, many studies have been carried out to overcome these shortcomings of PDT therapy using nanodrug delivery systems with positive results. Nanodrug delivery system-mediated PDT therapy can not only solve the shortcomings of PDT therapy, but more importantly, it can enhance the immune response generated by the combination of PDT therapy and ICB therapy. Combined treatment can further improve the therapeutic effect on tumors and expand the scope of the clinical treatment of tumors. Wang et al. [66] reported an antitumor immunotherapy through serum albumin-loaded photosensitizer PcM combined with PD-1/PD-L1 blockade therapy, which solved the deficiency of poor PDT tumor targeting ability by nanodrug delivery systems (Fig. 4A). The nanodrug delivery system-mediated combination of PDT with αPD-1 antibody had the highest primary tumor suppression rate (Fig. 4B), and the lowest tumor volume and weight (Fig. 4C and D). More importantly, in the 4T1 delayed distant primary tumor model, the abscopal effect of combination therapy was more obvious, as the highest inhibition rate of the delayed distant primary tumor (up to 59.21%) was more evident in the combination therapy group (Fig. 4E). Using flow cytometry to analyze CD8^+^ T cells, PD-1 and myeloid-derived suppressor cells (MDSCs) in TME after distal tumor treatment, the results showed that the combination therapy group had the highest percentage of CD8^+^ T cells, the highest PD-1 expression, and the lowest ratio of MDSCs, suggesting that nanodrug delivery systems enhanced the systemic antitumor adaptive immunity generated by the combined application of PDT and ICB therapy (Fig. 4F). When evaluating the systemic antitumor immune effects, in addition to the delayed distant primary tumor model described above, Huang et al. [67] found that nanodrug delivery systems combined with PDT and ICB therapy significantly increased the ratio of effector memory T cells (Tem) and central memory T cells (Tcm) in the spleen of mice, achieving long-term protection by activating the immune system (Fig. 4G and H).

### Nano-PTT drug delivery systems combined with ICB therapy to amplify the antitumor immune response

PTT produces heat therapy through a photothermal agent under the irradiation of NIR light, which causes tumor cell apoptosis and tumor ablation. PTT works well in treating local tumors but cannot effectively prevent tumor recurrence and metastasis [68]. One strategy to overcome the shortcomings of PTT is to combine it with immunotherapy by activating the body’s own antitumor immune system against metastatic tumors and residual tumor cells [69]. PTT can release many TAAs after tumor elimination, thus activating the body’s immune system. However, a single PTT cannot induce an effective antitumor immune response due to the influence of immune checkpoints and other factors [70]. After PTT ablates tumors, ICB therapy can solve the problem of tumor recurrence and metastasis, while nanodrug delivery systems can serve as carriers for the combination of the 2 therapies, and simultaneously promote the antitumor immune response generated by the combined therapy.

**Fig. 5.**
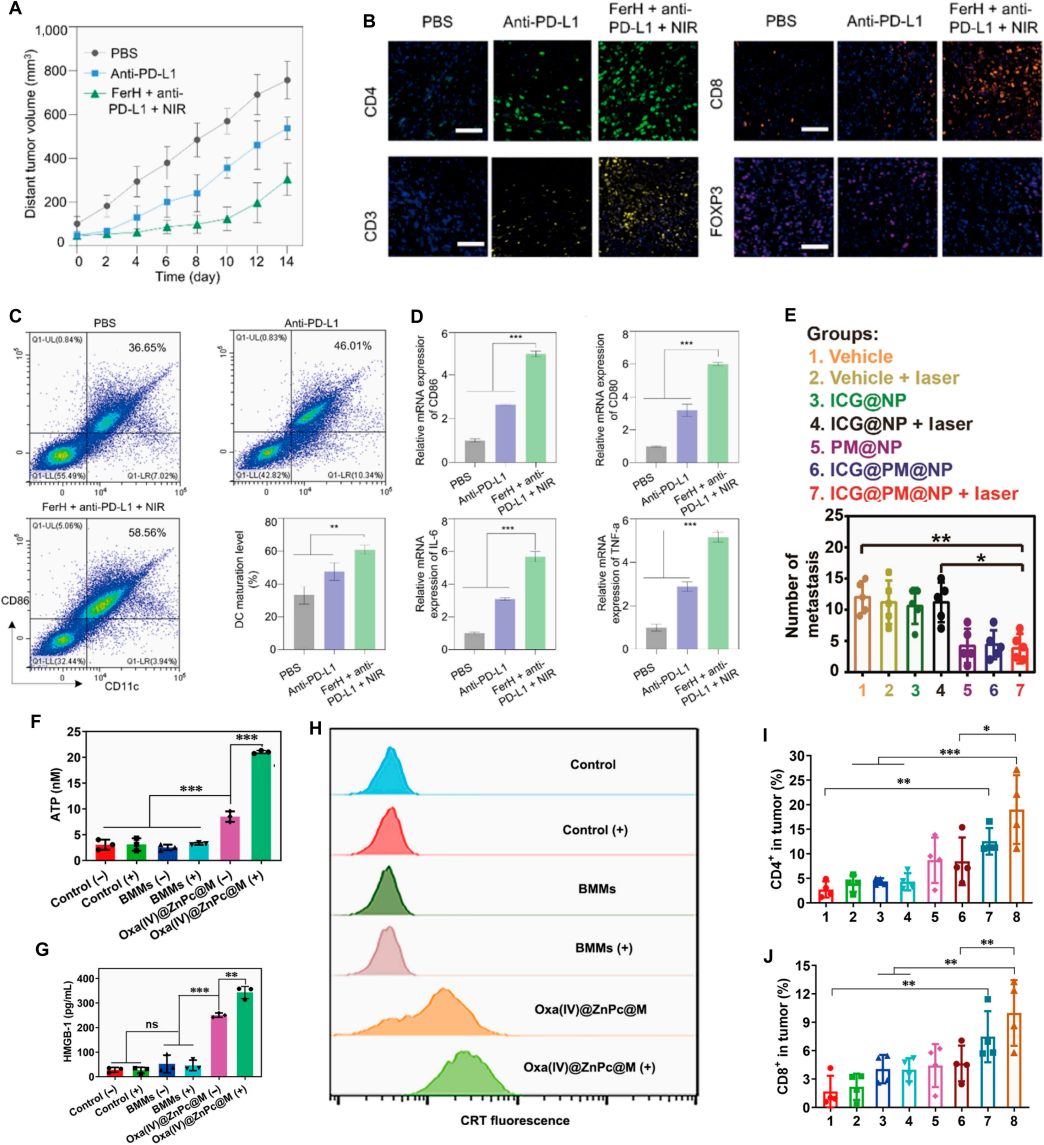
Nano-PTT drug delivery systems combined with ICB therapy to amplify the antitumor immune response. (A) The volume of distant tumors from the 4T1 tumor-bearing mice. Data are presented as mean ± SD. (*n* = 5). (B) Immunofluorescence investigation of distant tumor tissues after treatment with the following markers: CD3, CD4, CD8, FOXP3 (green: CD4, yellow: CD3, orange: CD8, pink: Treg, blue: nuclei). Scale bar, 50 μm. (C) Flow cytometry analysis of CD11c and CD80 (gated on CD11c^+^) in tumor-draining lymph nodes after different treatments and quantitative analysis of in vivo DC maturation level, experiments performed in triplicate with similar results (*n* = 3). (D) Relative mRNA expression in DCs and serum, including CD80, CD86, TNF-α, and IL-6 (*n* = 3). For (C) and (D), statistical significance was calculated via 1-way ANOVA with Tukey’s multiple comparisons (*P < 0.05, **P < 0.01, ***P < 0.001). Adapted with permission from [71], copyright 2022 Theranostics. (E) The number of 4T1 metastatic foci in the lungs of Balb/C mice on day 26 (*n* = 5). Statistical analysis was performed via the 2-tailed Student *t* test. *P < 0.05; **P < 0.01; ***P < 0.001. Adapted with permission from [72], copyright 2021 Journal of Nanobiotechnology. Quantitative determination of (F) adenosine triphosphate secretion (***P < 0.0001, ***P < 0.0001) and (G) HMGB-1 release of 4T1 cells after various treatments (*n* = 3, ***P < 0.0001, **P = 0.0017). (H) Flow cytometric examination of CRT exposure on the surface of 4T1 cells (*n* = 3). (I) CD4^+^ T cells (**P = 0.0060, ***P = 0.0002, *P = 0.0102) and (J) CD8^+^ T cell in tumors analyzed on day 6 after various treatments (**P = 0.0047, **P = 0.0038, **P = 0.0098). Adapted with permission from [74], copyright 2021 Nature Communications.

Based on the strategy of combined PTT and ICB therapy, He et al. [71] prepared the nanocluster FerH by self-assembling Fe^3+^ with HMT (a natural polyphenol), which significantly improved the retention time and infiltration depth of the photosensitizer in the tumor region through nanodrug delivery systems, and had a significant effect on tumor killing. FerH could produce powerful ICD effects by NIR irradiation and promote DCs maturation after killing tumor cells in vivo. After synergistic effects with anti-PD-L1, the combined therapy had a more obvious inhibition effect on the distal tumor, and the tumor inhibition rate reached 59.8% (Fig. 5A). Immunofluorescence staining was used to detected the infiltration of T cells, and the results showed that CD3^+^, CD4^+^ and CD8^+^ T cells had stronger fluorescent signals in the combined treatment group, indicating that the infiltration of T cells in tumors was significantly increased (Fig. 5B). Combined treatment also greatly increased the maturation of DCs, with notably higher expression of CD80 and CD86 (Fig. 5C and D). In addition, the expression of IL-6 and TNF-α also increased significantly (Fig. 5D), suggesting that the combined therapy based on nanodrug delivery systems amplified the antitumor immune response. In another article, Zhou et al. [72] developed an albumin-phenformin-based NP to block the PD-1/PD-L1 pathway by loading PM (a metformin analog) to induce adenosine 5′-monophosphate-activated protein kinase phosphorylation to reduce PD-L1 expression in tumor cells, achieving PD-1/PD-L1 blockade and solving the problem of tumor recurrence and metastasis after PTT treatment (Fig. 5E) [72]. Cano-Mejia et al. [73] triggered PTT by cytosine-phosphate-guanine motifs (CpG) oligodeoxynucleotide-coated Prussian blue nanoparticles (CpG-PBNPs), which showed significantly improved antitumor effects and were able to provide long-term effective antitumor immune protection to the organism when combined with CTLA-4 blocking antibodies. Huang et al. [74] suggested an all-in-one macrophage strategy and they constructed a nanodrug delivery system loaded with oxaliplatin prodrug and photosensitizer, which was transported to TME through macrophages. NIR light triggered photosensitizer-induced PTT combined with oxaliplatin prodrug-induced CT to kill tumor cells, while activating the ICD effect to enhance the antitumor response (Fig. 5F to H), which significantly increased the ratio of CD8^+^ T cells and CD4^+^ T cells in TME (Fig. 5I and J). After combination with anti-PD-L1, this strategy can significantly eliminate primary and bone metastatic tumors.

### Nano-CT drug delivery systems combined with ICB therapy to amplify antitumor immune response

CT is a treatment that uses chemically synthesized drugs systemically or locally to inhibit DNA synthesis, nucleic acid synthesis, protein synthesis, and other pathways of cancer cells to control tumor growth or kill tumor cells effectively. At present, CT is a common method for tumor treatment, but it has many disadvantages. Firstly, most chemotherapeutic drugs cannot target specific cells, which leads to the indiscriminate killing of normal cells and tissues and causes severe side effects. Secondly, CT produces definite immunosuppressive effects, and tumors are more resistant to antitumor immunity after CT. For example, CT promotes the secretion of IL-18 by macrophages, increases the expression of CD47 (the immune checkpoint of osteosarcoma), and promotes the immune escape of tumor cells [75]. Finally, CT has limitations in controlling the metastasis and escape of tumors, and single CT often cannot effectively prevent tumor metastasis and recurrence.

There are many limitations of single CT to cure tumors, and it is difficult to achieve the desired therapeutic outcome. Notably, nanodrug delivery systems have great potential to address these issues. Firstly, nanodrug delivery systems can solve the problem of poor tumor targeting of CT drugs by actively or passively targeting tumors. Qiao et al. [76] reported a type of bionic metal-organic skeletal nano-microspheres with erythrocyte membranes encapsulating TGFBR1 and the chemotherapeutic drug adriamycin, which achieved massive accumulation of chemotherapeutic drugs in tumor tissues through nanodrug delivery systems. Secondly, nanodrug delivery systems can reduce the toxicity of chemotherapeutic drugs and do not affect the efficacy of chemotherapeutic drugs. Weng et al. [77] utilized water-soluble pH-dependent antioxidant nanomaterials to deliver chemotherapeutic drugs, which reduced the damage of chemotherapeutic drugs to the kidney without affecting the antitumor efficacy. Tian et al. [78] reported a nanoantidote composed of dendrimer molecular wrapped by reduced cysteine, which significantly reduced the toxicity of chemotherapeutic drugs. In the cure of glioblastoma, this nano-antidote was only distributed in normal tissues and did not cross the blood–brain barrier, thus not affecting the tumor-killing effect of the chemotherapeutic drug temozolomide (TMZ, the glioblastoma standard CT), while capturing TMZ and significantly reducing the toxic side effects of TMZ on other normal tissues. Wu et al. [79] constructed a nanocomposite hydrogel to deliver chemotherapeutic and immunotherapeutic drugs. This nanocomposite hydrogel degraded in TME over time after injection, greatly reducing the toxicity of chemotherapeutic drugs to other normal tissues.

**Fig. 6.**
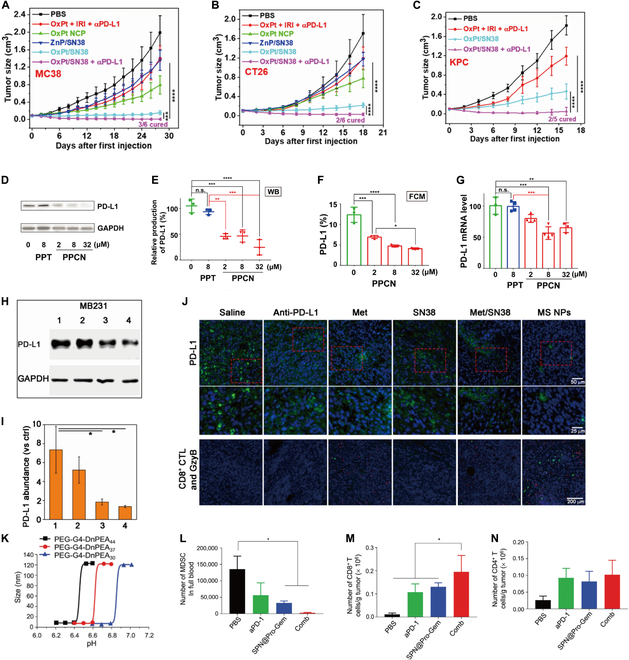
Nano-CT drug delivery systems combined with ICB therapy to amplify antitumor immune response. (A) Tumor growth curves of MC38 tumor-bearing C57BL/6 mice after indicated treatments, *n* = 6. (B) Tumor growth curves of CT26 tumor-bearing BALB/c mice after indicated treatments, *n* = 6. (C) Tumor growth curves of KPC tumor-bearing C57BL/6 mice after indicated treatments, *n* = 5. PBS, OxPt plus IRI, OxPt NCP, ZnP/SN38, or OxPt/SN38 was intravenously dosed once every 3 d with 3.5 mg/kg OxPt or and 6.2 mg/kg SN38 equiv to the MC38 model (8 doses) and CT26 and KPC models (6 doses). Adapted with permission from [82], copyright 2022 ACS Nano. (D and E) Western blot analysis of PD-L1 production in PPT- and PPCN-treated H460 cells. (F) Quantification of PD-L1 production in PPCN-treated H460 cells by flow cytometry. (G) Quantification of PD-L1 mRNA by reverse-transcription quantitative real-time polymerase chain reaction analysis. Data in (E) to (G) are means ± SDs, *n* = 3; *P < 0.05; **P < 0.01; ***P < 0.001; ****P < 0.0001; n.s., not significant. Adapted with permission from [83], copyright 2022 ACS Nano. (H) Western blot image of changes in PD-L1 level from MS NPs-treated MB231 cells. (Lane 1: MB231 cells, lanes 2 to 4: MB231 cells treated with different concentrations of MS NPs (20, 40, and 80 μg/ml respectively). (I) Densitometric analysis of PD-L1 bands. (J) Immunofluorescence staining of PD-L1 (green) (upper row), the magnified images (bottom row) and CD8^+^CTLs (red) and GzyB (green). Adapted with permission from [84], copyright 2021 Theranostics. (K) pH-Dependent size change of mPEG-G4-DnPEA44, mPEG-G4-DnPEA37, and mPEG-G4-DnPEA30 analyzed by dynamic light scattering (DLS). (L) Numbers of MDSCs in blood. (M) Numbers of CD8^+^ T cells per gram of the tumor upon various treatments. (N) Numbers of CD4^+^ T cells per gram of the tumor upon various treatments. Adapted with permission from [85], copyright 2021 Small.

Despite the immunosuppressive effects of CT, it has also been shown that CT also has certain immunostimulatory effects, such as cyclophosphamide (which inhibits the transcription of tumor cell DNA into RNA) which induces immunogenic death of cancer cells, triggers the immune system, and activates DCs and T cells when treating tumors [80]. Single CT is not sufficient to induce a strong immune response to resolve tumor metastasis and recurrence. However, by combination with ICB therapy through nanodrug delivery systems, it can induce a strong systemic antitumor immune response and effectively prevent tumor cells from escaping [81]. Jiang et al. [82] improved the delivery of the chemotherapeutic drug OxPt/SN38 through nanodrug delivery systems. Although it could significantly inhibit tumor growth and trigger strong ICD effects, it also up-regulated the expression of PD-L1 in tumor cells. The combination of this nanodrug delivery system with αPD-L1 produced a stronger antitumor immune response to eliminate subcutaneous MC38, CT26, and KPC tumors (Fig. 6A to C). In addition to being used in combination with immune checkpoint inhibitors, some nanomaterials themselves can also play a part in blocking immune checkpoint pathway. For example, Wang et al. [83] reported a kind of lipid bilayer NPs self-assembled by podophyllotoxin and poly (butyl acrylate) (PBA), which enhanced the delivery of the CT drug podophyllotoxin and significantly reduced its systemic toxicity [83]. Most importantly, the LNPs can significantly reduce the expression of PD-L1 while inducing tumor cell apoptosis and successfully combine CT and ICB therapy (Fig. 6D to G). Cai et al. [84] developed a nanodrug delivery platform (MS NPs) by assembling metformin (Met) and 7-ethyl-10-hydroxycamptothecin (SN38) through hydrogen bonding and electrostatic interactions, which effectively improved chemotherapeutic drug delivery [84]. Met can induce PD-L1 degradation in tumor cells and act as a PD-1/PD-L1 pathway blocker in the treatment system. MS NPs significantly reduced in the expression levels of PD-L1 in tumor cells through Met and inhibited tumor cell metastasis, especially lung metastasis, in vivo (Fig. 6H and I). The immunofluorescence staining results showed that the number and activity of CD8^+^ T cells in the tumors of tumor-bearing mice mediated by MS NPs were greatly increased, and the effect of immunotherapy was obviously enhanced (Fig. 6J). Tong et al. [85] reported a polymeric NP—SPN@Pro-Gem with a particle size of 120 nm at neutral pH and transformed to small particles of approximately 10 nm in the slightly acidic TME (Fig. 6K). SPN@Pro-Gem could pass through the tumor mesenchyme and deliver the chemotherapeutic drug gemcitabine (Gem) to the tumor parenchyma, enhancing the effect of CT in treating tumors. The combination of SPN@Pro-Gem with PD-1 antibody resulted in further improvements in tumor suppression and, more importantly, in increased levels of T cells during immunotherapy: a decrease in the number of MDSCs in the blood (Fig. 6L); and an effective increase in the number of CD4^+^ T cells and CD8^+^ T cells in the tumor (Fig. 6M and N). Zhu et al. [86] developed a lung-targeting nanodrug delivery system, Lip-CExo@PTX, based on a bispecific CRA-T cell-derived exosome that targeted mesothelin and programmed death ligand-1, loaded with chemotherapeutic agent paclitaxel (PTX). Lip-CExo@PTX enhanced the chemotherapeutic effects of PTX by delivering it to the tumor cells at lung tissues. At the same time, the anti-PD-1 single-chain antibody on the surface of exosomes prevented T-cell depletion by blocking the PD-1/PD-L1 pathway. Lip-CExo@PTX significantly prolonged the survival time of CT26 metastatic lung cancer model mice, which provided a prime example for the combination of CT and ICB therapy.

### Nano-RT drug delivery systems combined with ICB therapy to amplify the antitumor immune response

RT is a type of therapy that is used to treat local tumors by radiating tumor cells with radiation (such as x-rays and α, β, and γ rays generated by radioisotopes) to cause cell apoptosis, autophagy, necrosis, etc. [87]. RT is currently a common way for tumor treatment in clinic. It can be used to control local tumors well and promote the immunogenic death of tumor cells, release tumor neoantigens, and activate the antitumor immune system [88]. However, this immunogenic antitumor response induced by radiotherapy is weakened by tumor tissue through up-regulation of PD-L1 expression and immune escape of tumor cells, so it is difficult to induce a strong systemic immune response with single radiotherapy, and it cannot overcome and reduce the escape and metastasis of tumor cells [89]. In addition, radiotherapy can also weaken the immune system, enhance immune suppression, and damage the immune function of NK cells and T cells [87]. Therefore, radiotherapy needs to be combined with other therapies, such as ICB therapy, to minimize or avoid adverse effects on the immune system.

**Fig. 7.**
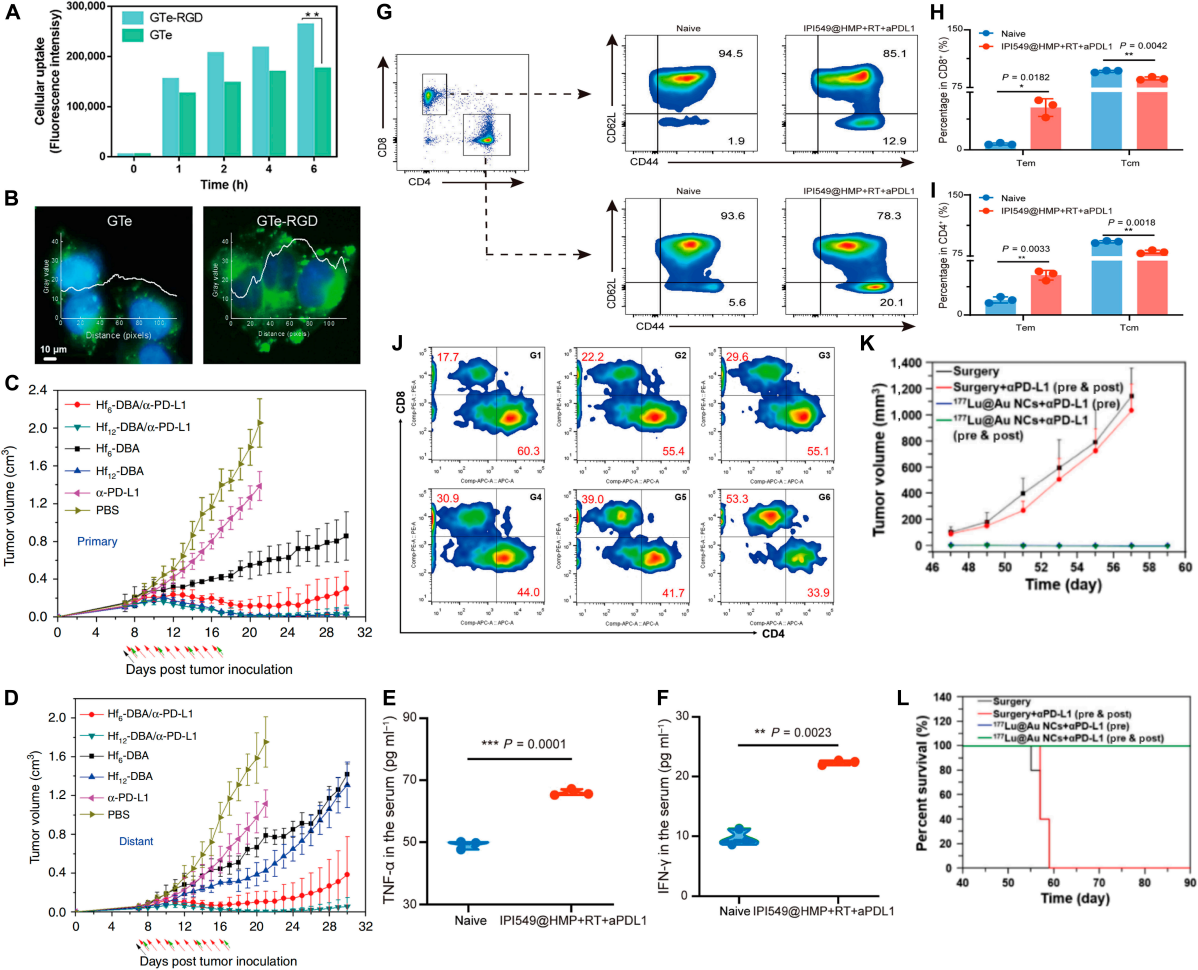
Nano-RT drug delivery systems combined with ICB therapy to amplify the antitumor immune response. (A) Quantitative analysis for uptake of GTe/GTe-RGD (1.25 mg/l) in A375 cells. (B) Typical images of A375 cells incubated with GTe and GTe-RGD for 6 h, and relative fluorescence intensity of the NPs quantified by ImageJ software. Adapted with permission from [92], copyright 2020 Matter. (C and D) Tumor growth curves of (c) primary tumors and (d) distant tumors of CT26 bilateral tumor-bearing mice treated with Hf6-DBA (with or without anti-PD-L1 antibody), Hf12-DBA (with or without anti-PD-L1 antibody), anti-PD-L1 antibody, or PBS with x-rays irradiation. Adapted with permission from [93], copyright 2018 Nature Communications. (E and F) Cytokine levels of TNF-α (E) and IFN-γ (F) in the serum after tumor rechallenging. (G to I) Representative flow cytometric analysis (G) and relative quantification of Tcm (CD62L^+^CD44^+^) and Tem (CD62L^−^CD44^+^) subset from CD8^+^ (H) and CD4^+^ (I) T cells in the spleen. Data were expressed as means ± SD (*n* = 3). Adapted with permission from [94], copyright 2022 Nature Communications. (J) Representative flow cytometry plots showing different types of T cells in the secondary tumors from different groups 10 d post treatment (CT26 tumor model). (G1: Surgery, G2: Surgery + αPD-L1, G3:99mTc@Au NCs [i.t.], G4:177Lu@Au NCs [i.t.], G5: 99mTc@Au NCs [i.t.] + αPD-L1, G6: 177Lu@Au NCs [i.t.] + αPD-L1). (K and L):Tumor growth (K) and survivorship curves (L) of mice in rechallenged tumor experiments after 40 d of the first tumors eliminated by surgery, surgery + αPD-L1 and 177Lu@Au NCs (i.t.) + αPD-L1. Adapted with permission from [95], copyright 2021 Nano Today.

While killing tumor cells, radiotherapy can induce immunogenic death of tumor cells, increase the release of TAAs and the expression of damage-related molecular patterns, and induce a stronger systemic antitumor immune response after being combined with ICB therapy [90]. The combination therapy of radiotherapy and ICB therapy mediated by nanodrug delivery systems can not only enhance the effect of radiotherapy in directly killing tumors but also activate the immune system. Nanodrug delivery systems can achieve precise radiotherapy and induce stronger immune responses through active or passive targeted delivery of radiotherapy drugs [91]. Huang et al. [92] synthesized tellurium nanostars (GTe-RGD) to achieve precise targeting of tumors by modifying RGD peptides on their surface. GTe-RGD significantly improves the uptake capability of tumor cells and has an extremely strong killing ability for tumor cells after x-ray irradiation (Fig. 7A and B). Ni et al. [93] reported a nanometallic organic framework based on the radiation enhancer HfO_2_ that cooperated with radiotherapy and ICB therapy to fight tumors. The combination therapy mediated by nanometallic organic framework significantly improved the antitumor effect in vivo, killing the primary tumor and significantly improving the control of the distal tumor (Fig. 7C and D). For the 2 combination therapies mentioned above, nanodrug delivery systems not only played the roles of targeted drug delivery and enhanced radiotherapy treatment but also enhanced the systemic antitumor immune response, which was essential to solve the problem of tumor cell escape. Guan et al. [94] constructed a nanodrug delivery system IPI549@HMP loaded with PI3-kinase γ via PEGylated HMnO2, which reduced tumor recurrence in combination with radiotherapy and ICB therapy. IPI549@HMPs were dissociated in the acidic TME, and then the nanomaterial carrier decomposed endogenous H_2_O_2_ to overcome the hypoxic properties of TME, and its loaded drug PI3-kinase γ down-regulated immunosuppressive myeloid cells to overcome immune tolerance. When combined with radiotherapy, IPI549@HMPs reduced the immunosuppression of the tumor and enhanced the sensitivity of TME to ICB therapy. The treatment results showed that after IPI549@HMPs were combined with radiotherapy and anti-PD-L1 to eliminate primary tumors, secondary tumors did not grow with the challenge of reinoculating tumors, and all mice survived for a long time with significantly increased secretion levels of TNF-α and IFN-γ (Fig. 7E and F). The transition of CD8^+^ and CD4^+^ T cells to Tcm and Tem was detected in the long-term surviving mice, suggesting that the combined treatment induced a systemic antitumor immune response and established immune memory (Fig. 7G to I). Pei et al. [95] reported that 2 radionuclide-labeled gold nanoclusters, 99mTc@Au NCs and 177Lu@Au NCs, induced ICD effects to promote the maturation of DCs and thus improve the antitumor immune response in vivo after radiotherapy treatment. Nanoclusters showed an excellent synergistic effect with αPD-L1, solving the problem of high PD-L1 expression in tumor cells after RT, and the percentage of intratumoral CTLs significantly increased to 53.3% (Fig. 7J). Compared with conventional surgical resection of tumors, the combination therapy showed better performance in the prevention of tumor recurrence, with little tumor growth and a significantly higher survival rate in the CT26 secondary tumor model mice treated with the nanodrug delivery system (Fig. 7K to L). In addition to these examples mentioned above, Zhang et al. [96] coupled Toll-like receptor agonist TLR7/8a with radiosensitive peptide hydrogel (Smac-TLR7/8 hydrogel) and prepared the nanofiber hydrogel Smac-TLR7/8 hydrogel by self-assembly, which also provided us a strategy to enhance immunotherapy by inducing macrophage polarization to the M1 type.

Because of the poor therapeutic effect of single ICB therapy for tumor treatment, nano drug delivery systems have the most examples of ICB therapy in combination with other treatment modalities, with a wide range of applications for both inorganic or organic nanomaterials (Table 3). For single ICB therapy, similar to cytokine therapy, researchers preferred to choose organic nanomaterials with better compatibility to enhance the immunotherapeutic effect. As for the combination of ICB therapy with PDT or PTT, the excellent photothermal conversion properties of inorganic metal nanomaterials have attracted the attention of many researchers. It can not only improve the efficiency of PDT and PTT in ablating tumors but also release plenty of TAAs, which greatly promote the activation of T cells. For the combination of ICB therapy and CT, researchers are more interested in organic nanomaterials. In addition to excellent biodegradability, the surface of nanomaterials such as PEG/PLGA has a lot of groups that can be modified. By modifying RGD peptides or other groups on the surface of these nanodrug delivery systems, it is possible to achieve precise targeting to tumor tissues, which greatly reduces the toxicity of CT. For the combination of ICB therapy with RT, more researchers are choosing inorganic nanomaterials to construct nano drug delivery systems due to the radiosensitizing effect of materials such as Hf. In general, in the study of the combined application of ICB therapy with other therapeutic approaches, nanodrug delivery systems not only improve the drug delivery efficiency and enhance the therapeutic effect but also significantly reduce the immunosuppressive characteristics of TME, which can enhance the therapeutic effect of ICB therapy and amplify the antitumor immune response.

**Table 3.** Nanodrug delivery systems in ICB therapy

Combination therapy type	Nanomaterials type	Nanocarrier	Load treatment drugs	Cancer model	Immune cell type	Therapeutic outcome	References
ICB therapy	Organic	MPC	Anti-PD-L1	LCPN	T cells	Realized glioma microenvironment-responsive blood–brain barrier-crossing delivery of ICB antibodies and significantly prolonged animal survival	[53]
ICB therapy	Organic	PLGA-PEG	TGFβR1 inhibitors	B16F10	T cells	Delayed tumor growth and extended the survival of mice harboring colorectal tumors	[54]
			TLR7/8 agonist	MC38			
ICB therapy	Organic	PPCs	IAP antagonists	B16F10	T cells	Significantly inhibited the growth of primary and secondary tumors	[56]
ICB therapy	Organic	PLGA-lecithin-PEG	Vitamin C/ICG	MB49	T cells	Polarized protumor M2 type macrophages to antitumor M1 type macrophages and promoted the infiltration of CTLs in the tumor	[60]
					Macrophages		
ICB therapy	Organic	pIC/Zn^2+^	pIC	CT26	T cells	Controlled tumor growth in syngeneic mouse models of colorectal cancer and a spontaneous mouse model of prostate cancer	[61]
				MC38			
				LL2			
ICB therapy	Organic	PD-L1 nanobody	TLR7 agonist	CT26	T cells	Enhanced tumor immunogenicity and converted “cold” tumors into “hot” tumors by activating innate immunity and promoting the activity of antigen-presenting machinery within the tumors	[62]
					DCs		
ICB therapy and PDT therapy	Organic andinorganic	Albumin	PcM	4T1	T cells	Significantly increased tumor suppression and promoted distal tumor regression	[66]
ICB therapy and PDT therapy	Organic andinorganic	CaO_2_/HA	CuS	4T1	T cells	Improved immunotherapy efficacy and long-term protection effect for body	[67]
				CT26			
ICB therapy and PTT	Self-assembly	HMT/Fe^3+^	HMT	4T1	T cells	Accelerated immune activation and substantially augmented the therapeutic effects for the distant tumors	[71]
					DCs		
ICB therapy and PTT	Organic	Albumin-phenformin	ICG/PM	CT26	T cells	Effectively inhibited tumor metastasis	[72]
				4T1			
ICB therapy and PTT	Inorganic	Prussian blue	CpG	Neuro 2a	T cells	Completely regressed primary and secondary tumors	[73]
					NK cells		
ICB therapy and CT	Self-assembly	Zn^2+^/DOPA	Oxaliplatin prodrug ZnPc	4T1	T cells	Eliminated primary and bone metastatic tumors	[71]
					Macrophages		
ICB therapy and CT	Organic andinorganic	TMS/DOPC/	OxPt/SN38	MC38	T cells	Inhibited tumor growth on subcutaneous, spontaneous, and metastatic tumor models	[82]
					DCs		
		DSPE-PEG_2000_		CT26	Macrophages		
ICB therapy and CT	Self-assembly	PBA	Podophyllotoxin	H460	T cells	Significantly inhibited tumor growth and extended survival	[83]
ICB therapy and CT	Self-assembly	Met/SN38	Met/SN38	MB231	T cells	Significantly improved survival rates	[84]
				4T1			
				Luc-4T1			
ICB therapy and CT	Organic	PEG	Gemcitabine	Panc02	T cells	Increased tumor suppression and improved TME	[85]
ICB therapy and CT	Organic	CAR-T cell-derived exosomes	PTX	CT26	T cells	Significantly improved survival rates	[86]
ICB therapy and RT	Organic and inorganic	GSH	Te	A375	T cells	Clearly increased the survival time	[92]
				HeLa	DCs		
		RGD peptide		4T1	Macrophages		
ICB therapy and RT	Inorganic	HfO_2_	Hf_6_-DBA/Hf_12_-DBA	CT26	T cells	Significantly eliminated primary tumors and suppressed distal tumors	[93]
					NK cells		
					DCs		
ICB therapy and RT	Organic and inorganic	PEG/MnO2	PI3-kinase γ	CT26	T cells	Completely eliminated primary tumors, inhibited secondary tumors, and extended survival time	[94]
					Macrophages		
ICB therapy and RT	Inorganic	GSH-Au	Technetium-99m	4T1	T cells	Significantly suppressed the growth of spontaneously metastatic tumors and lengthened the survival time of the transgenic mice	[95]
			lutecium-177	CT26	DCs		

## Nanodrug Delivery Systems in Tumor Vaccines

Tumor vaccination is an active immunotherapy method that mainly regulates the immune system through TAAs, tumor peptides, DNA, RNA, etc., and induces the body to produce a tumor-specific immune response, especially tumor-specific T-cell immune response to prevent or treat tumors. Currently, sipuleucel-T and talimogene laherparepvec [T-VEC] are the 2 therapeutic vaccines approved by the US and the European Union to treat prostate cancer and melanoma separately [97]. The composition of tumor vaccines includes antigens and immune adjuvants. The antigen needs to be immunogenic to activate the antitumor immune system mediated by T cells, and the immune adjuvant can further enhance the immune response [97,98]. Immune adjuvants can stimulate and enhance the immune response by improving antigen presentation or other ways to solve the problem of low immunogenicity of tumor antigens and not enough to trigger a strong enough immune response. In addition, adjuvants can also avoid immunosuppression, so adding immune-modified adjuvants to cancer vaccines is an effective way to overcome immunosuppression obstacles [99]. At present, in the research on improving the delivery of tumor vaccines in vivo and enhancing antitumor immune response, various nanodrug delivery systems have been used as carriers of the cancer vaccine to induce stronger antitumor immune response [100]. Nanodrug delivery systems enhance the immune response to tumor vaccines mainly by the following 3 mechanisms. Firstly, the targeted characteristics of the nanodrug delivery system allow for precise targeting of tumor vaccines, which effectively protect antigens and adjuvants from premature degradation before reaching the site of action [101]. Secondly, when some nanomaterials are used to constitute tumor vaccines, they can participate in activating and enhancing antitumor immune responses due to their adjuvant properties [102]. Finally, nanodrug delivery systems formed by the self-assembly of antigens and adjuvants can induce a stronger antitumor immune response in vivo.

### Nanodrug delivery systems improve the presentation of tumor vaccines in vivo and enhance targeting capability

TAAs applied in tumor vaccines are autoantigens, which exist not only in tumor cells but also in normal human cells, so the human body will develop certain tolerance to these antigens, making it difficult to induce a vigorous immune response. In addition, traditional tumor vaccines are ineffective in clinical treatment due to their low efficiency of antigen encapsulation capacity, poor lymph node targeting performance, and weak lysosomal escape ability [103]. Therefore, the precise targeting property of nanodrug delivery system-mediated tumor vaccines is important for successful tumor vaccine design. Tumor vaccines based on nanodrug delivery systems can not only efficiently encapsulate antigens and adjuvants through electrostatic interactions, hydrophobic interactions, and covalent binding and protect antigens and adjuvants during vaccine delivery but also precisely target lymph nodes or immune cells and efficiently release them in APCs based on their specific physical or chemical properties [103–105].

**Fig. 8.**
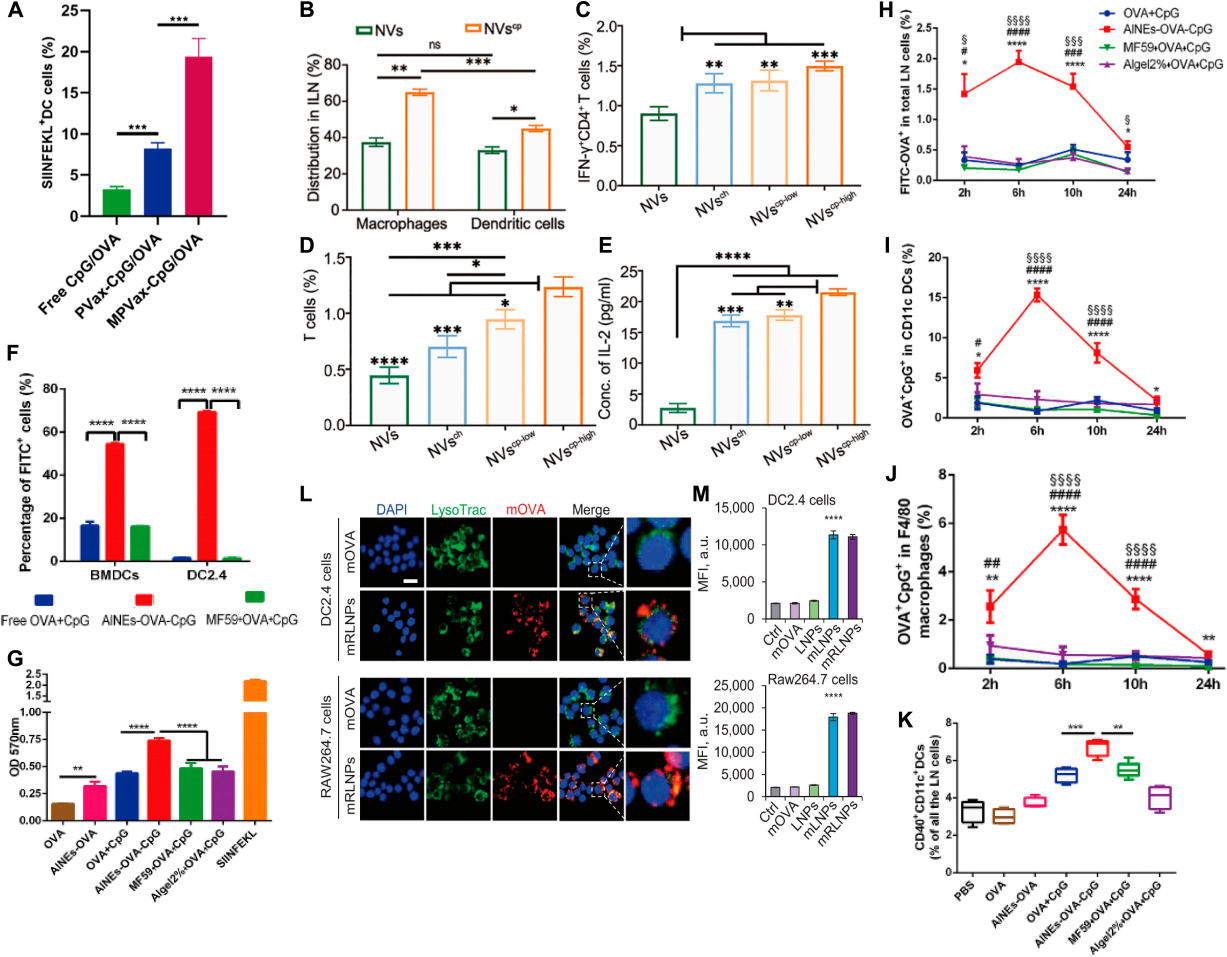
Nanodrug delivery systems improve the presentation of tumor vaccines in vivo and enhance targeting capability. (A) Antigen cross-presentation analysis of BMDCs after coculture with free CpG/OVA/, PVax-CpG/OVA and MPVax-CpG/OVA for 24 h and staining with antimouse H-2Kb bound to SIINEFKL antibody. (ns: not significant, *P < 0.05, **P < 0.01, ***P < 0.001). Adapted with permission from [108], copyright 2022 Biomaterials. (B) NVs and NVscp distribution in DCs and 826 macrophages in ILN. (C and D) Flow cytometry was used to determine the percentages of OVA specific (C) IFN-γ-producing CD4^+^ cells and (D) IFN-γ-producing CD8^+^ T cells. (E) The release of IL-2 from splenocytes of immunized C57BL/6 mice after ex vivo restimulation with OVA 257-264 peptide (SIINFEKL) was determined using ELISA. Adapted with permission from [109], copyright 2021 Biomaterials. (F) Uptake efficiency of AlNEs-OVA-CpG (containing FITC-labeled OVA) by BMDCs and DC2.4. (G) AlNEs-OVA-CpG significantly promoted cross-presentation of OVA on BMDCs. (H) Percentages of FITC-OVA^+^ cells in total lymph nodes cells. (I and J) Percentages of OVA- and CpG-positive cells in DCs (I) or macrophages (J) of the popliteal lymph nodes cells. (K) Percentage of CD40^+^CD11c^+^DCs in total lymph node cells at day 3 after administration. Adapted with permission from [110], copyright 2022 Journal of Controlled Release. (L) Representative confocal images of DC2.4 and RAW264.7 cells incubated with Cy5.5 labeled mOVA and mRLNPs for 6 h, showing cellular uptake and endo/lysosome escaping of mOVA. The endo/lysosomes were stained with Lyso Tracker Green (green). Scale bar: 20 μm. (M) Cellular uptake of DC2.4 and RAW264.7 cells incubated with saline, mOVA, LNPs, mLNPs, and mRLNPs for 6 h determined with flow cytometry. Adapted with permission from [116], copyright 2022 Advanced Functional Materials.

**Fig. 9.**
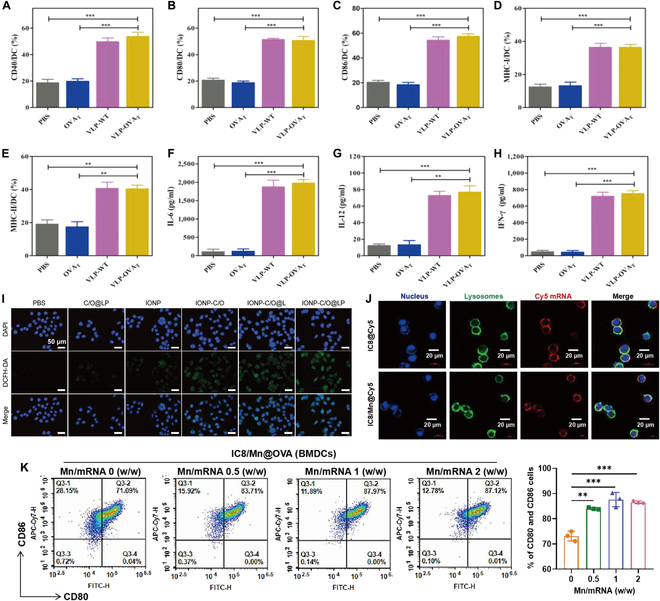
The adjuvant properties of nanodrug delivery systems can amplify the antitumor immune response. (A to E) BMDCs were incubated with PBS, OVAT, VLP-WT, or VLP-OVAT for 24 h and then stained with antibodies to assess surface markers including CD40 (A), CD80 (B), CD86 (C), MHC-I (D), and MHC-II (E) by FACS. (F to H) Supernatants were collected for analysis of the expression of IL-6 (F), IL-12 (G), and IFN-γ (H) by ELISA. Scale bars, 20 μm. Data are expressed as the mean ± SEM (*n* = 3) (*P < 0.05; **P < 0.01; ***P < 0.001). Adapted with permission from [120], copyright 2021 Biomaterials. (I) Representative fluorescence microscopy images of ROS expressed by DCs after incubation with different nanoformulations. Intracellular ROS was stained by 2′7′-dichlorodihydrofluorescein diacetate (DCFH-DA). Adapted with permission from [121], copyright 2022 Advanced Materials. (J) Confocal fluorescence images of DC2.4 cells incubated with IC8@Cy5 and IC8/Mn@Cy5 in 10% fetal bovine serum (FBS) medium for 5 h. DAPI (4′,6- diamidino-2-phenylindole; blue) was used to track the cell nuclei, and Lyso Tracker Green DND-26 (green) was used to track lysosomes; red fluorescence was from Cy5 mRNA. (K) Flow cytometry examination of BMDCs maturation (CD11c^+^CD80^+^CD86^+^) 24 h after incubation with IC8@OVA and IC8/Mn@OVA. FITC, fluorescein isothiocyanate. Adapted with permission from [122] , copyright 2022 Science Advances.

DCs are specialized APCs in the human body that can capture tumor cells after apoptosis and cross-present TAAs to CD8^+^ T cells [106,107]. DC vaccination is an immunological approach that targets DCs and induces DCs maturation, followed by activation of CD8^+^ T cells through cross-presentation of DCs to induce a strong antitumor immune response in vivo. The effective delivery and subsequent activation of antigens to DCs are major challenges in the development of DC vaccines, and nanodrug delivery systems not only enable the targeting ability of tumor vaccines but also enhance lysosomal escape and thus improve the efficiency of antigen cross-presentation [102]. Xu et al. [108] reported a polymeric pathogen-like nanovaccine (MPVax) with mannan as the shell and poly (lactic acid)-poly(ethyleneimine) (PLA-PEI) assembled NPs as the core that adsorbed the tumor antigen protein ovalbumin (OVA) and Toll-like receptor 9 (TLR9) agonist CpG by electrostatic interactions. MPVax significantly improved the recognition ability of DCs to vaccines by modifying mannans on the surface, which in turn improved the presentation efficiency of nanovaccines to DCs. Compared to NPs without mannans, the presentation efficiency of DCs was increased 2-fold and compared to free CpG/OVA, the presentation efficiency was increased 6-fold (Fig. 8A). Su et al. [109] reported a nanovaccine (NVs^cp^) made by growing calcium peroxide in situ based on OVA self-assembly, which significantly improved the cross-presentation efficiency of exogenous antigens. NVs^cp^ activated the NADPH oxidase 2 complex by forming lipid peroxidation products on the surface, promoting vaccine escape via endo-/lysosomal lipid peroxidation and drainage into the lymph nodes, thereby enhancing cross-presentation to lymph node DCs (Fig. 8B). In addition to DCs, NVs^cp^ also significantly increased the uptake and presentation of vaccines by macrophages. Flow cytometry and enzyme-linked immunosorbent assay (ELISA) were used to detect T cells in the spleen of mice injected with NVs^cp^ and their IL-2 secretion after in vitro activation. The results showed that compared with mice injected with NVs, the percentages of IFN-γ^+^CD4^+^ T cells and IFN-γ^+^CD8^+^ T cells in the spleen of mice injected with NVs^cp^ were much higher, and IL-2 secretion was also significantly higher, and the results were best in the NVs^cp-high^ group (Fig. 8C to E), suggesting that NVs^cp^ induced an effective antitumor immune response in vivo. Chen et al. [110] constructed an AlNEs-OVA-CpG nanovaccine by adsorbing aluminum hydroxide on the surface of the oil-in-water emulsion MF59 and wrapping antigen (OVA) and immune enhancer (CpG). The in vitro results showed that the uptake efficiency of DC2.4 cells and bone marrow-derived cells (BMDCs) on AlNEs-OVA-CpG and cross-presentation efficiency to APCs were much higher than those of free OVA/CpG, and the uptake efficiency of DC2.4 cells on AlNEs-OVA-CpG was 45 times higher than that of free OVA/CpG (Fig. 8F and G). Notably, other normal cells (human umbilical vein endothelial cell and murine L929 cells) showed low uptake efficiency of AlNEs-OVA-CpG, which suggested a strong targeting ability of AlNEs-OVA-CpG. The nanovaccine also significantly improved the targeted delivery efficiency to lymph nodes, with colocalization experiments showing that nanodrug delivery systems improved the presentation efficiency of the vaccine to lymph node DCs in vivo by 17.37-fold and macrophages by 29.29-fold (Fig. 9H to K).

In addition to improving the delivery of tumor antigen vaccines, nanodrug delivery systems can also serve as carriers for nucleic acid vaccines, improving their delivery behavior in vivo. Nucleic acid vaccines are mainly divided into DNA vaccines and mRNA vaccines. Although DNA vaccines can stimulate the body’s immune response much better, there is a risk of genetic mutation. In contrast, mRNA vaccines do not carry the risk of gene mutation, as they do not integrate into the genome, and they also have the advantages of high efficiency and are economical, so they have been widely researched [111,112]. mRNA vaccines stimulate an immune response by delivering messenger RNA sequences to the body that produce proteins similar to tumor antigens. Nanodrug delivery system-mediated delivery of mRNA vaccines can effectively protect nucleic acids and improve mRNA stability. LNPs are currently FDA-approved mRNA carrier materials with several advantages, such as simple formulation, high mRNA payload capacity, good biocompatibility, and they have important advantages in both cell-mediated and humoral immunity activation in vivo [113]. Furthermore, LNPs can protect mRNA from degradation, facilitate intracellular delivery, and promote the endosomal escape of mRNA cargo [114]. For example, Hashiba et al. [115] developed a novel branched ionizable lipid that significantly improved mRNA stability and delivery efficiency when contained in LNPs. LNP-mediated delivery of mRNA vaccines could greatly improve the delivery efficiency of mRNA and without toxicity or unnecessary immunogenicity, making LNPs ideal carriers for developing mRNA tumor vaccines currently. LNPs have made good progress as nonviral delivery carriers to deliver mRNA to the cytoplasm, but the storage and transport of mRNA-LNP vaccines have limited their clinical applications. To solve the problem of storage and transportation, Jia et al. [116] constructed an mRNA nanovaccine using LNPs as a carrier for mRNA that achieved nanovaccine preservation by cross-linking with a hyaluronan dynamic hydrogel. This nanodrug delivery system significantly improved the stability of the mRNA vaccine. Western-blot data showed that compared to free mOVA-treated DC2.4 and RAW264.7 cells, the nanodrug delivery system-mediated mOVA-treated cells showed high expression of OVA, indicating that this nanodrug delivery system-mediated mOVA was not degraded (Fig. 8L and M). All of the above examples showed that nanodrug delivery systems could not only improve the delivery efficiency of tumor vaccines but also help preserve tumor vaccines, which have excellent clinical application prospects. In addition to vaccination applications, mRNA can be used to target immune cells in the vivo to achieve antitumor effects.

### The adjuvant properties of nanodrug delivery systems can amplify the antitumor immune response

In addition to improving the delivery and targeting of tumor vaccines, some biomaterials can be used as immune adjuvants to activate the immune system. Virus-like particles (VLPs) are natural nanomaterials derived from various plant viruses, phages and mammalian viruses that can bind to tumor-specific antigens by chemical/peptide chain binding and constitute nanovaccines. The nanovaccine can effectively enter the lymph nodes and be phagocytosed by DCs after local injection, activating the major histocompatibility complex-I (MHC-I) signaling pathway and achieving effective antigen presentation [117,118]. Apart from delivery and targeting, VLPs can also act as immune adjuvants, binding to CD209 on DCs and inducing the release of cytokines IFN-γ and TNF-α to produce robust cellular immune responses [119,120]. Alam et al. [119] constructed a series of mannoside ligands by modifying VLPs (derived from phage Qβ), among which VLPs modified by aryl mannoside induced maturation of DCs and expression of proinflammatory cytokines characteristic of the Th1-like immune response. When constructing nanovaccines, these aryl mannoside-modified VLPs could not only serve the function of targeting DCs but also participate in inducing an antitumor cell immune response and play the role of an immune adjuvant, which has good application prospects. Li et al. [120] combined the B and T epitopes (OVA_B_ and OVA_T_) of the model antigen OVA with the C segment of VLPs (derived from phage P22), respectively, and self-assembled them with scaffolding protein to form 2 nanovaccines, VLP-OVA_B_ and VLP-OVA_T_. VLP-OVA_B_ induced strong antibody titers (>10^5^) and a Th1-type immune response, while VLP-OVA_T_ significantly increased the percentages of CD4^+^, CD8^+^ and effector T cells and decreased the percentage of MDSCs in tumors or spleens in vivo. In this antitumor research, in addition to performing the role of targeting antigen delivery, VLPs also showed that the expression of CD40, CD80, CD86, MHC-I, and MHC-II on the cell membranes of BMDCs induced by blank VLPs and the concentrations of IL-6, IL-12, and IFN-γ in the supernatant were not significantly different from those of VLP-OVA_T_, suggesting that VLPs themselves could be used as immune adjuvants to induce the activation of BMDCs (Fig. 9A to H).

In addition to the above VLPs, other nanomaterials can also serve as immune adjuvants in the construction of nanovaccines and participate in the stimulation of antitumor immune responses in vivo. Chen et al. used MF59 and aluminum hydroxide to construct a kind of nanodrug delivery system in which aluminum hydroxide was involved in coating antigens and adjuvants and acted as a Th2-type immune adjuvant to enhance the immune response [110]. Meng et al. [121] constructed a water-soluble nanomaterial IONP (iron oxide nanoparticle) by replacing the oleic acid ligand of Fe_3_O_4_ particles with DIP-PEG-MAL. Then, IONPs were attached to CpG and OVA and wrapped with a lipid membrane of the DC-targeted cyclic peptide P30 to generate the nanovaccine IONPC/O@LP. IONPC/O@LP could deliver OVA and CpG to the cytoplasm and lysosomes of immature DCs and enhance the activation of DCs. Notably, the nanomaterial IONPs not only imparted nanovaccine detectability by magnetic resonance imaging but also generated ROS in cells with good adjuvant effects (Fig. 9I). In addition to tumor antigen vaccines, adjuvant properties of nanomaterials can also enhance the immune response of mRNA tumor vaccines in vivo. Fan et al. [122] constructed an mRNA nanodrug delivery system (IC8/Mn LNPs) with high immunogenicity by adding Mn to the novel ionizable lipid IC8. The addition of Mn endowed this nanodrug delivery system with adjuvant properties, which enhanced mRNA expression by promoting lysosomal escape and stimulated the maturation of APCs by activating the STimulator of Interferon Genes (STING) pathway (Fig. 9J and K). The results showed that the best antigen presentation of APCs was achieved when Mn/mRNA = 1:1.

### Self-assembly of tumor antigens and adjuvants to form nanovaccines can induce stronger antitumor immune responses

**Fig. 10.**
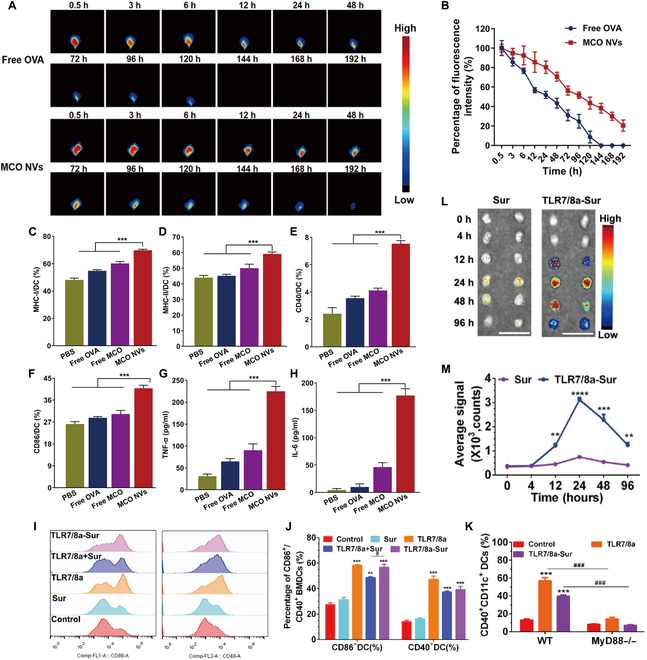
Self-assembly of tumor antigens and adjuvants to form nanovaccines can induce stronger antitumor immune responses. (A) Antigen retention in Free OVA and MCO NVs formulations at the injection site detected by the in vivo fluorescent imaging system. (B) Quantitative analysis of the percentage of fluorescence intensity at different time points. (C to H) The expression levels of (C) MHC I, (D) MHC-II, (E) CD40, and (F) CD86 were measured with flow cytometers. The cytokine secretion levels of (G) TNF-α and (H) IL-6 were measured with ELISAs. Data were expressed in the form of mean ± SD (*n* = 6). ***P < 0.001. Adapted with permission from [123], copyright 2021 Nano Letters. (I) Representative flow cytometry histograms and (G) percentages of CD86 and CD40 stimulated with TLR7/8a-Sur nano vaccine or other indicated formulations. (K) The percentage of CD40^+^CD11c^+^ DCs detected by flow cytometry. (L) Representative images and (M) quantification of fluorescence signals in inguinal lymph nodes on the left and right sides excised from mice immunized with different vaccine formulations at scheduled time points. Data are expressed as mean ± SDs. *P < 0.05, **P < 0.01, and ***P < 0.001, compared with the control using 1-way ANOVA. Adapted with permission from [124], copyright 2022 Acta Biomaterialia.

Except for the above 2 ways to enhance the immune response of nanovaccines, antigens and adjuvants are essentially small molecules such as proteins or peptides, which can form nanovaccines by self-assembly. Through self-assembly, nanovaccines can change the distribution behavior of the original drug, improve the targeting effect of antigens and adjuvants, enrich the drug at the target site, increase the local drug concentration, reduce off-target effects, and reduce immunotoxicity continuously and effectively [21]. The self-assembly approach can significantly improve the loading efficiency, simplify the preparation process, and enable efficient delivery of antigen and adjuvant in vivo [123,124]. Liu et al. constructed an amphiphilic molecule, MPLA-CpG, from monophosphatidyl A (MPLA, a kind of TRL4 agonist) and CpG, and then self-assembled it with OVA to form nanovaccines, MCO NVs [123]. In vivo imaging system results showed that MCO NVs significantly extended the antigen retention time of APCs, from 120 to 192 h (Fig. 10A and B). This self-assembled nanovaccine activated DCs, raised the expression of MHC-I, MHC-II, CD40, and CD86 and increased the levels of TNF-α and IL-6 compared with the control group (Fig. 10C to H). Song et al. [124] self-assembled TLR7/8 agonist-epitope conjugate into nanostructures in an aqueous solution and achieved the self-assembly of antigen and adjuvant to constitute the nanovaccine TLR7/8a-Sur by directly chemically linking the antigen Sur peptide. Compared with free TLR7/8a and Sur peptide, TLR7/8a-Sur induced greater activation of DCs and a higher proportion of CD86^+^ DCs and CD40^+^ DCs, and enhanced antigen uptake and endosome escape (Fig. 10I to K). In vivo imaging system results showed that the self-assembled nanovaccine continued to accumulate in inguinal lymph nodes and showed good targeting ability and extended antigen retention time compared to the free Sur peptide (Fig. 10L and M).

For tumor vaccine therapy, organic nanomaterials are used more widely (Table 4). The final destination of antigens or adjuvants loaded by nanodrug delivery systems is the lymph nodes or other immune organs, thus demanding a higher level of biocompatibility for nanomaterials. In addition, researchers choose some nanomaterials with adjuvant properties such as VLPs more to construct nanodrug delivery systems, so as to enhance the immune response induced by tumor vaccines. Self-assembly of antigens and adjuvants to construct nanodrug delivery systems is also an approach that researchers frequently choose to solve the problem of tumor vaccine delivery and thus enhance immunotherapy. With the further research on tumor vaccines, inorganic metal ions such as Mn^2+^ and Zn^2+^ have been proved that they have the effect in activating the immune system in vivo, so many researchers add some inorganic metal ions to construct nanodrug delivery systems to induce stronger immune response. In general, nanodrug delivery systems can be designed to load tumor vaccines, efficiently package antigens and adjuvants, and improve the colocalization, biodistribution, and presentation of vaccines with APCs in vivo. Whether it is the targeting and presentation of nanodrug delivery systems or their adjuvant properties, these ultimately induce stronger antitumor immune response in vivo.

**Table 4.** Nanodrug delivery systems in tumor vaccines

Nanomaterials type	Nanocarrier	Load treatment drugs	Cancer model	Immune cell type	Therapeutic outcome	References
Organic	PLA-PEI	OVA/CpG	B16-OVAMC38	DCs	Elicited robust immune response in vivo	[108]
Inorganic	Calcium peroxide	OVA	B16-OVA	DCsT cells	Significantly increased the ratios of intratumoral CD8^+^ T/regulatory T cells and achieved prominent tumor therapy effects.	[109]
Inorganic	Aluminum hydroxide/MF59	OVA/CpG	EG7-OVA	DCs	Significantly inhibited tumor growth and prolonged mice survival	[110]
Organic	LNPs	mOVA/R848	B16F10-OVA	DCs	Efficiently delivered mRNA encoding tumorantigens to DCs	[115]
Organic	VLPs	IVT mRNA	ID8	Macrophages	Induced antitumor immunity and promoted tumor regression	[116]
Organic	VLPs	Qβ-Man540	LLC	DCs	Elicited DCs maturation and induced the expression of the proinflammatory cytokines	[119]
Organic	P22 VLPs	OVA_B_/OVA_T_	EG.7-OVA	DCs	Significantly inhibited tumor growth	[120]
Inorganic	IONPsP30 peptide	OVA_P_/CpG	B16-OVA	DCs	Effectively increased the antigen-specific T cells in both tumor and spleen, inhibited tumor growth	[121]
Self-assembly	MPLA	OVA/CpG	EG.7-OVA	DCs	Inhibited the tumor growth and prolonged the survival of tumor-implanted mice.	[123]
Self-assembly	TLR7/8a-epitope	Sur/MAGE-1/gp100	B16	DCs	Resisted the invasion of B16 cancer cells and produced excellent therapeutic effects against established B16 melanoma tumors	[124]

## Nanodrug Delivery Systems in ACT therapy

ACT therapy, also known as cellular immunotherapy, includes chimeric antigen receptor-modified T cells (CAR-T cells), NK cells, tumor-infiltrating lymphocytes (TILs), T-cell receptor modified T cells (TCR-T cells), etc. It is a type of immunotherapy in which autologous or allogeneic immune cells are isolated, activated, or genetically modified in vitro to expand a sufficient number of immune cells with antitumor activity and are reinfused back into cancer patients to amplify the cellular immune function in patients. Compared with traditional therapies, cellular immunotherapy has the advantages of good efficacy, low toxic side effects, strong targeting, and memory immunity. It can not only directly eliminate the tumor but also activate the body’s immune function to play a role in inhibiting the tumor, effectively overcoming the tumor immune escape mechanism to keep tumors in a relative dynamic balance in its unique TME. These immune cells are mainly some cells that promote antitumor immune response such as T cells, B cells, and NK cells. After genetic modification in vitro, these immune cells enhance the ability to target and kill cancer cells and activate the body’s immune system. In clinical applications, genetically modified immune cells have shown good results alone or in combination with other therapeutic methods [125]. However, the antitumor activity of ACT therapy is highly dependent on the proliferation and persistent activity of premetastatic cells, and the therapeutic efficacy of ACT therapy, especially for solid tumors, is greatly limited due to a series of potential factors in vivo, such as immunosuppression of the TME, poor persistence of T cells, and T-cell function impairment [126,127].

ACT therapy faces challenges in clinical application due to its systemic side effects, and the rapid development of nanodrug delivery systems offers new strategies to overcome its limitations. When applied to ACT therapy, nanodrug delivery systems can not only promote the expansion and activation of adoptive cells in vitro but also promote tumor infiltration of adoptive cells or edit antitumor T cells in vivo [19,128]. For different types of ACT therapy, nanodrug delivery systems can improve the immune response induced in vivo in different ways, and we will introduce the application of nanodrug delivery systems in CAR-T therapy and other ACT therapy (NK cell therapy, TIL therapy, and CAR-M1 therapy) in the following examples.

### Nanodrug delivery systems in CAR-T therapy

CAR-T therapy focuses on isolating T cells from patients’ blood, genetically modifying them in vitro to enhance their ability to target and kill cancer cells, and then infusing them back into their bodies to multiply and kill cancer cells [129]. Using T cells to target tumors usually solves problems such as weak signals released from dead tumor cells, lack of T-cell recognition due to the absence of MHC class I molecules in tumor cells, deficiency of T-cell infiltration in tumors, and T-cell dysfunction, so CAR-T therapy aims to activate or enhance the antitumor immune system in the body [130]. After artificial construction, CAR-T cells can recognize various cell surface structures. For example, the surface of CAR-T cells is composed of outer parts used for antigen recognition cells and has strong tumor cell recognition ability, which makes CAR-T cells independent of the presentation of MHC class I molecules, greatly enhancing their targeting and killing ability to tumor cells [131]. In addition to targeting tumor cells, CAR-T cells can also be used for other cells. Tisagenlecleucel and axicabtagene ciloleucel are 2 CAR-T products that have been approved to target CD19 cells for the treatment of relapsed diffuse large B-cell lymphoma, both of which have achieved excellent therapeutic results [132–134]. Moreover, idecabtagene vicleucel (Abecma), which targets B-cell maturation antigens for the treatment of myeloma, has been approved by the FDA [135].

CAR-T therapy has achieved remarkable results in antitumor therapy, but the serious toxicity and side effects that are associated with the application of CAR-T therapy should also be considered. The 2 most common toxic and side effects are cytokine release syndrome (CRS) and immune effector cell-associated neurotoxicity syndrome. CAR-T cells release large amounts of perforin and cytokines after recognizing antigens, leading to tumor scorching (inflammatory cell death), and the released cytokines, such as IL-2, also activate macrophages, releasing inflammatory factors. All of these processes ultimately lead to the overactivation of immune effector cells and proinflammatory cytokines over normal physiological levels, which triggers CRS [132]. Inflammation caused by CAR-T cells can also lead to injury or even death of endothelial cells in the blood–brain barrier, which in turn destroys the blood–brain barrier, allowing immune cells and cytokines to penetrate, leading to neuronal damage and dysfunction and triggering immune effector cell-associated neurotoxicity syndrome [135]. Therefore, when treating with CAR-T therapy, the impacts of the therapy’s toxic side effects need to be considered. Another limitation of the application of CAR-T-cell therapy is its high production cost. The isolated T cells require specialized facilities such as viral vectors to deliver CAR genes into the T cells so that the T cells can express CAR proteins. In addition, the production rate of CAR-T cells limits their use in some rapidly developing tumors.

**Fig. 11.**
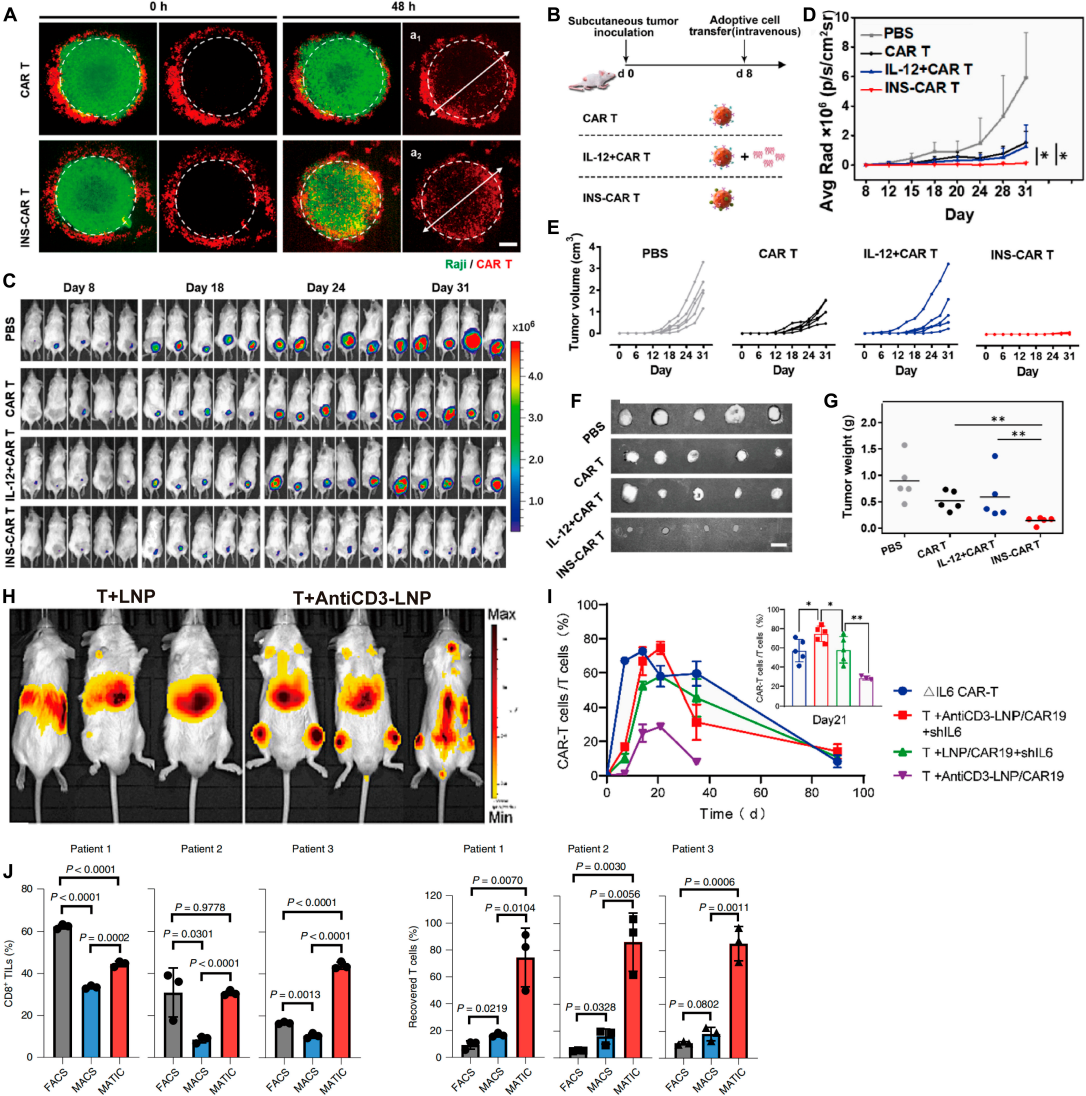
Nanodrug delivery systems in adoptive cell therapy. (A) Infiltration of INS-CAR T and CAR T cells. Raji cell spheroids (anti-CD19-PE, green) were incubated with CAR T cells or INS-CAR T (APC-conjugated anti-CD3, red) for 48 h, and images were recorded with the confocal quantitative imaging cytometer CQ1. The tumor spheroid is marked with a white dotted circle. (B) Experimental scheme. Luci-Raji tumor-bearing mice were intravenously injected with CAR T cells (1 × 10^7^ cells), IL-12^+^CAR T cells (0.25 μg of IL-12, 1 × 10^7^ cells) or INS-CAR T (0.25 μg of IL-12, 1 × 10^7^ cells) on day 8 post tumor inoculation. (C and D) Tumor-bearing mice were monitored by bioluminescence imaging (C) and quantified by detection of the radiance in the region of interest over a course of 31 d (D). Bioluminescence imaging units: Avg Rad ×10^6^ (p/s/cm2/sr). (E) Individual tumor growth curves were constructed by measuring tumor volume using calipers at the indicated time points. (F and G) Photographs and weights of excised tumors at the end of experiments. Data are presented as the means and standard errors. Significance was calculated using 1-way ANOVA with Tukey’s posttest, *n* = 5. *P < 0.05, **P < 0.01. Adapted with permission from [140], copyright 2022 Biomaterials. (H) Biodistribution of fluorescent CD3 antibody targeting or nontargeted LNPs 24 h after tail-vein injection. Data are from 5 mice per treatment condition. The mice on the left were administered LNP/ CAR19 + shIL6, and the mice on the right were administered AntiCD3-LNP/CAR19 + shIL6. The CD3 antibody modified the LNPs targeting the lymphoid tissue in which the T cells were located. (I) The number of CAR-T cells produced by LNPs in vivo. The number of CAR-T cells in T + AntiCD3-LNP/CAR19 + shIL6 group was significantly higher than that in other groups on the 21st day. Data are represented as mean ± SD of 5 mice in each group. Adapted with permission from [141], copyright 2022 Journal of Controlled Release. (J) Quantitation of the purity and recovery of FACS/MACS/MATIC for isolating human NSCLC samples. *P < 0.05, **P < 0.01, ***P < 0.001, log-rank test for survival curve, unpaired *t* test for bar plot, mean ± SD. Each dot represents a biological replicate. Adapted with permission from [150], copyright 2022 Journal of Controlled Release.

When cooperating with CAR-T therapy in the treatment of tumors, nanodrug delivery systems have the following 4 main advantages: (a) improving the transport of CAR-T cells in vivo, enhancing cytotoxicity and targeting; (b) reducing the toxicity and side effects of CAR-T in vivo; (c) shortening the cycle of CAR-T therapy for tumors; and (d) reducing treatment costs [136]. Hu et al. [137] used NPs to encapsulate IL-15 and delivered by nanofiber hydrogel to maintain the proliferation and activity for CAR-T cells, which could improve the therapeutic efficacy of CAR-T therapy. In 2017, Smith et al. developed DNA NPs to import CAR genes into the nucleus of T cells, enabling the production of CAR-T cells in vivo and achieving excellent antitumor effects [138]. Parayath et al. [139] reported an injectable nanocarrier that delivers in vitro-transcribed (IVT) CAR or TCR mRNA. Nanoparticles targeted CD8^+^ T cells by electrostatic adsorption of CD8 antibody on their surface and delivered mRNA to T cells, which induced T cells to express tumor-specific CARs. In mouse models of human leukemia, prostate cancer, and hepatitis B-induced hepatocellular carcinoma, NPs all showed excellent therapeutic results. In these methods of targeting the delivery of NPs loaded with specific genes to T cells in vivo, nanodrug delivery systems and CAR-T therapy have demonstrated good synergy, successfully performing in situ programming of T cells in the body and solving the problems of production cost and action cycle of CAR-T therapy. To address the weaknesses of CAR-T therapy in terms of killing and toxic side effects, Luo et al. [140] developed a human serum albumin NP loaded with IL-12 and conjugated this NP to CAR-T cells, which significantly strengthened the effect of CAR-T cells in treating solid tumors and reduced the toxic side effects. IL-12 could enhance the killing ability of T cells against tumor cells and significantly improve antitumor immune response. However, IL-12 has side effects such as proinflammatory toxicity, so it is necessary to accurately target tumor tissue. Based on human serum albumin NPs, Luo et al. designed an IL-12 nanostimulator (INS) and successfully prepared INS-CAR-T cells by biological hybridization with CAR-T cells. Incorporation of INS significantly enhanced the tumor infiltration ability of CAR-T cells (Fig. 11A). The high recognition ability of CAR-T cells to tumors solved the problem of targeted delivery of IL-12 to tumor tissue, greatly reducing the tumor load of tumor-bearing mice (Fig. 11B to D). After reaching tumor tissue, IL-12 improved the cytotoxicity of CAR-T cells, showing good synergistic effects. After injection of INS-CAR-T cells into the body and stimulation by Raji tumor cells, INS was isolated, and IL-12 was gradually released to promote the tumor-killing effect mediated by CAR-T cells, significantly reducing the tumor volume and weight (Fig. 11E to G). As another typical example, Zhou et al. constructed a LNP system loaded with IL-6 silencing RNA (shRNA) and CD19-CAR binding gene with surface-modified CD3 antibody, which could produce the same antitumor effect as conventional CAR-T in vivo while simplifying the CAR-T preparation process and reducing the CRS caused by CAR-T [141]. LNPs modified with CD3 antibody can significantly target lymph nodes and induce the highest number of specific CAR-T cells in vivo on the 21st day, with the same antitumor effect as CAR-T cells prepared in vitro, but with a significantly reduced preparation cycle (Fig. 11H and I). This nanodrug delivery system-based CAR-T therapy produced CD8^+^ CAR-T cells dramatically on days 14 to 21, and the number of CD8^+^ CAR-T cells was significantly decreased on days 35 to 90 after killing the tumor (Fig. 11I). In addition, under the influence of IL-6 shRNA, the toxicity of CRS was significantly reduced, improving the safety of CAR-T therapy in vivo. In the above example, nanodrug delivery systems down-regulated cytokine release by delivering silenced genes to improve the safety of CAR-T therapy. In addition to this approach, nanodrug delivery systems can also improve the safety of CAR-T therapy by accurately targeting tumor sites. Nguyen et al. [142] optimized the design of CAR-T cells and obtained LiCAR-T cells that respond to blue or NIR light. After binding to tumor antigens, LiCAR-T cells can kill tumor cells under the activation of blue or NIR light. Researchers further combined LiCAR-T cells with highly efficient upconversion nanoparticles, using nanoparticles as light sensors to convert NIR light with strong tissue-penetrating ability into blue light, thereby achieving activation of LiCAR-T cells in situ and precise targeting of cellular immune responses, avoiding side effects related to immunotherapy, and greatly improving the safety of CAR-T cells.

### Nanodrug delivery systems in other ACT therapy

In addition to improving the antitumor effect of CAR-T therapy, nanodrug delivery systems can also cooperate with other ACT therapy, such as NK cell therapy and TIL therapy, to improve the effect of immunotherapy in vivo. NK cells are a kind of cytotoxic lymphocytes that can kill tumor cells through various mechanisms such as secretion of perforin and granzyme, activation of immune cells to release chemokines and cytokines, and antibody-dependent cytotoxicity [143–145]. NK cell-based tumor immunotherapy has good tumor-killing effects and biosafety, but the lack of ligands on the surface of NK cells to target specific tumors leads to their insufficient tumor infiltration ability [145,146]. Nanodrug delivery systems can enhance the tumor infiltrating ability of NK cells in a nongenetic manner. Meng et al. [147] prepared N3-NK cells targeting tumors and modified IL-21-loaded NPs on their surface to obtain N3-NK-NPs, which significantly improved the tumor infiltration ability of NK cells. After injection into the body, N3-NK-NPs could specifically recognize and kill tumor cells, while NPs could continuously release IL-21 on the surface of NK cells, effectively stimulating NK cells while limiting their systemic toxicity.

TIL cell therapy is a highly personalized therapy in which TILs are isolated from tumor tissue and transfused back into the patient after culture and massive expansion in vitro [148]. The effector cells of TIL therapy are naturally selected and enriched, with a high percentage of tumor-specific T cells, which have the advantages of various targets, strong tumor infiltration ability, and few side effects compared with other therapies, thus having a promising application [148,149]. However, TILs in tumors are few and difficult to separate fluorescence-activated cell sorting (FACS), usually loses 50% to 70% of the input cells when sorting TILs, and magnetic-activated cell sorting (MACS) accumulates a large number of dead cells and debris in the chromatography column, resulting in poor purity of sorted TILs. Wang et al. [150] developed a microfluidic affinity targeting of infiltrating cells (MATIC) sorting method based on microfluidic technology for quantitative immunomagnetic sorting of TILs. This sorting method used magnetic nanoparticles conjugated with antibodies to label TILs, making TILs immunomagnetic. Compared to traditional cell sorting, TILs can be recovered up to 30 times with better tumor killing effects (Fig. 11J).

Macrophages are an important type of immune cell in our immune system, which not only can directly kill tumor cells but also have the function of antigen presentation. Kang et al. [151] used mannose-conjugated polyethylenimine (MPEI) as a nanocarrier loaded with CAR-IFN-γ-encoding plasmid DNA to target macrophages in vivo and convert macrophages into CAR-M1 with antitumor activity. These CAR-M1 cells were capable of CAR-mediated cancer phagocytosis, antitumor immunomodulation, and inhibition of solid tumor growth. After targeting macrophages, nanodrug delivery systems induced macrophage polarity toward toward M1 and significantly increased the presentation of tumor antigens to T cells, increasing the ratio of activated CD8^+^ T cells in tumors.

Compared to the other 3 immunotherapies, there are fewer reports on the combined application of nanodrug delivery systems and ACT therapy (Table 5). An important point is that ACT therapy is based on cells in transit, while nanodrug delivery systems are traditionally a system for drug delivery to treat disease, and traditional ways of thought limit the combined application of these 2 therapeutic approaches. The above examples demonstrate that nanodrug delivery systems can contribute to the antitumor immune response by serving in various aspects of ACT therapy, such as cell isolation, expansion, and enhanced in vivo targeting of adoptive cells. Among ACT therapy, CAR-T cell therapy is one of the current approaches that has been proved with high effectiveness in treating tumor, so in the example application above, nanodrug delivery system is mainly applied in CAR-T cell therapy. To deal with the problem of weak immune effect in CAR-T therapy, researchers employed nano drug delivery systems loaded with cytokines or immune adjuvants to bind on the surface of CAR-T cells to enhance their killing effect on tumor cells. For the systemic toxicity problem in CAR-T cell therapy, researchers have mostly used organic nanoparticles loaded with silencing RNA of relevant toxic cytokines (such as IL-6 shRNA, etc.) to reduce the relevant toxicity produced by CAR-T cell therapy. In addition, nano drug delivery systems are also able to load mRNA to generate CAR-T cells with strong antitumor effects in vivo. NK cell therapy and CAR-M1 therapy are similar to CAR-T cell therapy and researchers prefer organic nanomaterials to improve therapeutic efficacy and reduce toxicity. Besides organic nanomaterials, magnetic nanoparticles in inorganic nanomaterials have also been used by researchers for TIL therapy, greatly improving the sorting efficiency of TIL cells. Nanodrug delivery systems also have great prospect in TCR-T cell therapy, but there are no examples currently available of both being used together. In general, the synergistic antitumor effects of nanodrug delivery systems and ACT therapy are worthy of further investigation in future antitumor research work.

**Table 5.** Nanodrug delivery systems in ACT therapy

ACT therapy type	Nanomaterials type	Nanocarrier	Load treatment drugs	Cancer model	Immune cell type	Therapeutic outcome	References
CAR-T therapy	Organic	PBAE polymer	IVT mRNA	RajiLNCaP C42 HepG2	CAR-T cells	Induced sufficient host T cells expressing tumor-specific CARs or virus-specific TCRs to cause disease regression	[139]
CAR-T therapy	Organic	HAS	IL-12	Raji	CAR-T cells	Significantly boosted antitumor efficacy and minimized unwanted toxicity in vivo	[140]
CAR-T therapy	Organic	DLin-MC3-DMAcationic lipids	IL-6 shRNA and CD19-CAR	Raji-LucK562	CAR-T cells	Increased the convenience of CAR-T therapy and effectively reduce the CRS problem	[141]
CAR-T therapy	Organic	CRY2CIBN	CD19-CAR	B16K562Raji	CAR-T cells	Enabled both spatial and temporal control over T cell-mediated antitumor therapeutic activity in vivo with greatly mitigated side effects	[142]
NK cell therapy	Organic	NHS-SS-NHS	IL-21	Raji	NK cells	Greatly promoted proliferation and activation of NK cells while limiting systemic toxicity and side effects and significantly improved TME	[147]
TIL therapy	Inorganic	Magnetic nanoparticles	Anti-CD39	B16F10	T cells	Immunomagnetic cell sorting recovered up to 30-fold higher numbers of TILs, and the higher levels and diversity of the recovered TILs accelerated TIL expansion and enhanced their therapeutic potency	[150]
CAR-M1 therapy	Organic	MPEI	CAR-IFN-γ-encoding plasmid DNA	Neuro-2a	CAR-M1	Induced CAR-M1 macrophages in vivo for effective antitumor immunity and avoided the complex and costly processes of ex vivo CAR-cell manufacturing	[151]

## Conclusions

In summary, the combination of nanodrug delivery systems and immunotherapy is highly effective in the treatment of tumors. Nanodrug delivery systems can not only solve the delivery and targeting problems of immunotherapy drugs and minimize the toxicity and side effects of immunotherapy but also enhance the therapeutic effect of immunotherapy and amplify the antitumor immune response in vivo. Firstly, nanodrug delivery systems can precisely deliver cytokine based therapeutic drugs to the TME to enhance the function of immune cells and reduce their systemic toxic effects. Secondly, the combination of PDT, PTT, CT, RT, and other therapeutic methods mediated by nanodrug delivery systems with ICB therapy not only solves the problem that PDT and other therapeutic methods cannot avoid tumor recurrence and metastasis but also significantly improve the immunotherapeutic effect of ICB therapy and enhance the immune system’s control and killing of tumors, especially the killing effect of T cells. Thirdly, in the application process of tumor vaccines, nanodrug delivery systems can not only significantly improve the delivery of various vaccines but also serve as adjuvants to enhance the in vivo antitumor immune response induced by tumor vaccines. Finally, nanodrug delivery systems can enhance the therapeutic effect of ACT therapy in various ways, such as in vivo generation of CAR-T cells by loading CAR genes and in vitro sorting of TIL cells by magnetic nanoparticles. It can also reduce the systemic toxic side effects, such as cytokine storms produced by ACT therapy.

## Prospects

Immunotherapy is an innovative treatment approach to tumor treatment, with the advantage in activating the immune system and enhancing the body’s immune response to suppress the growth and metastasis of tumor cells. With the properties of targeting, excellent biocompatibility, and controlled release, nanodrug delivery systems could not only carry immunotherapeutic drugs to tumor but also amplify the antitumor immune response generated after treatment.

With researchers’ better understanding in immunotherapy, nanodrug delivery systems can not only be used in the above 4 immunotherapy treatments but also used in combination with these new immunotherapy treatments such as trained immunity to amplify the immunotherapy effect. “Trained immunity” is focused on creating durable antitumor innate immune response by inducing the training properties of myeloid progenitor cells. Nanomaterials, which themselves can interact with phagocytic myeloid cells, are ideal platforms for “trained immunity”. Priem et al. [152] developed a bone marrow-targeted nanodrug delivery systems, MTP10-HDL, which promoted “trained immunity” and induced durable antitumor response. The construction and application of MTP10-HDL demonstrated the potential of nanodrug delivery systems in combination with “trained immunity”. Besides novel immunotherapy on immune cells, nanodrug delivery systems can also promote the immunotherapeutic effect of new tumor cell immune death approaches such as cellular pyrolysis. For example, Zhong et al. [153] developed a nanoliposome (GM@LR), which was able to convert 4T1 apoptosis to pyroptosis, and Mn^2+^ that was used to construct the nanodrug delivery system was able to induce the STING pathway activation. GM@LR was able to significantly enhance immunotherapy and was an excellent example for combining cellular pyroptosis with nanomaterials.

There are more than 100 nanomedicines on the market or in clinical trials, and most of these nanomedicines are liposomes or lipid-based nanoparticles. It can be seen that nanomedicines prepared from organic nanomaterials such as liposomes are relatively easier to translate clinically. But some inorganic nanomaterials such as SiO_2_, Au etc. are also under clinical trials and have great potential for tumor imaging applications. Although these nanomedicines are currently focused on CT and radiotherapy, antitumor immune nanomedicines are increasingly emphasized with researchers deeply examining antitumor immunotherapy [154]. For different immunotherapy treatments, various nanodrug delivery systems can be used to promote tumor treatment.

Despite the great therapeutic efficacy of nanodrug delivery systems and immunotherapy in animal experiments, there are no nanomedicines on immunotherapy that have been marketed globally. The clinical translation of nanomedicines related to antitumor immunotherapy faces the following challenges. Firstly, while nanodrug delivery systems can improve the slow onset of immunotherapy, single immunotherapy is still too weak for solid tumor compared to traditional surgery, CT, or radiotherapy. This is particularly obvious in ICB therapy, so many researchers choose to avoid this problem by combining it with other therapy approaches. Secondly, the structure composition of nanomedicines is complex, and the synthesis methods are generally multistep reactions. These factors make it difficult to achieve controlled production for nanomedicines. Thirdly, immunotherapy-related nanomedicines’ biological evaluations in vivo and in vitro could not fully simulate the complex TME in human body, which lead to the lower-than-expected clinical therapy effect. Finally, many new immunotherapy-related nanomedicines use novel nanomaterials, but these materials do not have enough data on safety and biocompatibility research, which limits the clinical translation of nanomedicines.

Clinical translation challenges cannot mask the advantages and potential application of nanodrug delivery systems in antitumor immunotherapy. Currently, various nanomaterials have been gradually exploited and applied in antitumor immunotherapy with encouraging results. In the combination of immunotherapy and nanodrug delivery systems, researchers need to select different nanomaterials according to their different requirements. In this review, we describe the application of nanodrug delivery systems in 4 immunotherapy types, including cytokine therapy, ICB therapy, tumor vaccine therapy, and ACT therapy, and discuss different nanomaterials chosen by researchers for different immunotherapy types. Finally, we expect that this review can provide some helpful suggestions to researchers when developing nanomedicines or selecting nanomaterials.

## Ethical Approval

This article does not have any ethical issues and was approved by the Experimental Animal Ethics Sub-Committee of the Academic Committee of Beijing University of Traditional Chinese Medicine, Ethics No. BUCM-4-2021120801-4101.

## Data Availability

The authors declare that the data supporting the findings of this study are available within the paper and its Supplementary Materials files.
